# Ecological Roles and Shared Microbes Differentiate the Plastisphere from Natural Particle-Associated Microbiomes in Urban Rivers

**DOI:** 10.1021/acs.est.5c06538

**Published:** 2025-08-08

**Authors:** Yingyu Bao, Yuen-Wa Ho, Zhiyong Shen, Edmund Y. Lam, James K. H. Fang, Kenneth M. Y. Leung, Patrick K. H. Lee

**Affiliations:** † School of Energy and Environment, 53025 City University of Hong Kong, Hong Kong SAR, China; ‡ Department of Food Science and Nutrition and Research Institute for Future Food, 26680 The Hong Kong Polytechnic University, Hong Kong SAR, China; § State Key Laboratory of Marine Environmental Health, City University of Hong Kong, Hong Kong SAR, China; ∥ Department of Electrical and Electronic Engineering, 25809 The University of Hong Kong, Hong Kong SAR, China; ⊥ Department of Chemistry, City University of Hong Kong, Hong Kong SAR China; # Low-Carbon and Climate Impact Research Centre, City University of Hong Kong, Hong Kong SAR, China

**Keywords:** urban rivers, microplastics, plastisphere, ecological roles, microbial sharing

## Abstract

The “plastisphere,” comprising microbes associated with microplastics (MPs), may have substantial ecological impacts on riverine ecosystems. However, little is known about how the microbiomes associated with anthropogenic MPs compare with those associated with natural particles (NPs) in urban rivers with varying MP pollution levels. We therefore conducted a comparative analysis of the metagenomes associated with MPs and NPs (100–5000 μm) and river water (RW) across 10 urban river systems. Although we found similarities in taxonomic and functional compositions between the microbiomes associated with MPs and NPs, the plastisphere exhibited distinct associations with specialized taxa and life-history strategies. These unique traits enhanced the potential of the plastisphere for complex carbohydrate and plastic degradation, nitrate and nitric oxide reduction, and antibiotic resistance and virulence compared with the NP or RW microbiomes. Furthermore, MPs supported the sharing of unique microbes with the surrounding RW; these shared microbes possessed enhanced horizontal gene transfer capabilities and potentially could disperse traits of the plastisphere into the broader RW microbiomes. This study highlights the distinct ecological roles and shared microbes of the plastisphere, indicating that MP pollution may substantially and uniquely impact the function and health of riverine ecosystems.

## Graphical abstract

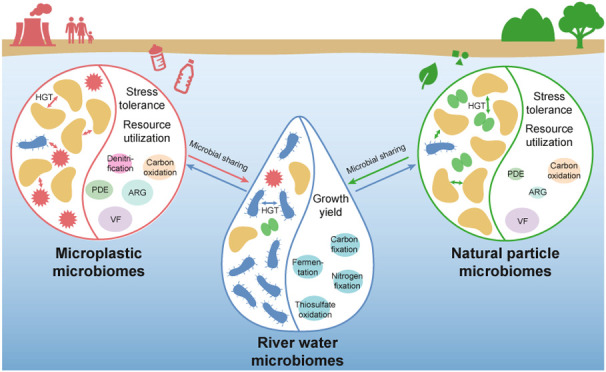

## Introduction

Urban rivers have long supported biodiversity and provided essential services for sustainable urban development.^1^ However, anthropogenic disturbances caused by various contaminants have threatened urban rivers’ ecological and social functionality worldwide.^2^ In recent years, microplastics (MPs) (i.e., synthetic plastic particles <5 mm in size^3^) have emerged as concerning anthropogenic pollutants.^4^ MPs from various sources are ubiquitous in urban rivers and transported to neighboring aquatic environments by water flows.^5–7^ Microbes, which respond sensitively to anthropogenic disturbances,^8^ play crucial roles in key biogeochemical processes within riverine ecosystems.^9^ When MPs enter urban rivers, microbes rapidly colonize their surfaces, forming biofilms known as the “plastisphere”^10^ that are closely tied to the fates and ecological impacts of MPs within riverine ecosystems.^11^

Riverine microbes can colonize not only anthropogenic MPs but also natural particles (NPs)^12^ composed of various materials (e.g., organic matter, inert minerals, and black carbon),^13^ which are ubiquitous in urban rivers. Once colonized, both particle types facilitate microbial aggregation and interactions and provide colonizing microbes with access to resources and protection from predators and environmental stresses.^14^ Other riverine microbes are free-living and suspended directly within the water column.^14^ These microbe types are not isolated within riverine ecosystems but are interconnected, with water–particle interfaces serving as microbial sharing hotspots.^15^ Such microbial sharing can influence the taxonomic and functional diversity and stability of receiving habitats.^16^ Horizontal gene transfers (HGTs) between microbes within receiving habitats can amplify impacts on riverine ecosystems by enabling functional gene spread via microbial sharing, potentially altering the metabolic capabilities of resident microbes.^17^

As MP pollution increases in urban rivers worldwide, these anthropogenic particles will increasingly provide surfaces for microbial colonization within riverine ecosystems. However, the similarities and differences between microbes attached to MPs versus those attached to NPs are often overlooked.^18^ Although MPs and NPs share many physical characteristics (e.g., size, shape, and density), MPs also possess unique properties, including specific polymer composition, leaching of chemical additives (e.g., plasticizers, colorants, and antioxidants), and high affinity for specific hydrophobic organic contaminants,^19^ which may influence the microbes they host and their interactions with river water (RW) microbiomes.

To investigate the ecological impacts of MPs on riverine ecosystem health and function under ecologically relevant conditions, we conducted a field-based metagenomic study in major urban rivers in Hong Kong, which are characterized by dynamic flow and varying levels of MP pollution. We collected and analyzed microbiomes from MPs and NPs (100–5000 μm), along with RW, from the same locations to ensure consistent environmental exposure. We compared their taxonomic and functional profiles, assessed their potential ecological roles in biochemical cycling, plastic degradation, antibiotic resistance, and virulence, and examined potential microbial sharing and HGT between MPs and NPs with RW. We hypothesize that the intrinsic properties of MPs selectively recruit microbes with both overlapping and distinct ecological roles compared with NPs, resulting in unique microbiome compositions that may differentially influence RW through microbial sharing and gene transfer.

## Materials and Methods

Two 50 L surface water samples were collected from each of the 15 sites across 10 major urban rivers in Hong Kong (Figure S1a), affected by varying levels of anthropogenic influence (Table S1), and filtered on-site using stainless-steel sieves. Retained particles (100–5000 μm) and filtered RW were used for (1) MP, NP, and RW microbiome analysis and (2) MP and NP characterization and quantification. Detailed procedures are provided in Text S1 and Figure S1b provides an overview of the sampling and analysis strategy. Briefly, for microbiome analysis, particles were first classified as MPs or NPs using nondestructive methods—visual sorting under a stereomicroscope (SMZ1270i; Nikon, Tokyo, Japan)—followed by confirmation with optical photothermal infrared (O-PTIR) spectroscopy (mIRage IR microscope; Photothermal Spectroscopy Corp., Santa Barbara, USA). Representative MP and NP microbiomes were obtained by randomly pooling at least 30 classified particles per water sample. RW microbiomes were collected on sterile 0.2-μm poly­(ether sulfone) filters (Pall Corporation, Port Washington, NY, USA) after filtering 0.5–1 L of RW through a 100-μm filter. For particle characterization and quantification, visually sorted NPs were enumerated by type and size under a stereomicroscope. A separate set of particles underwent chemical digestion for MP characterization, and the remaining particles were analyzed for polymer composition using μ-Raman spectroscopy (inVia confocal Raman microscope; Renishaw, Wotton-under-Edge, UK). MP shapes and sizes were determined via stereomicroscopy. The potential presence of plastic additives and aging in visually sorted MPs was assessed using O-PTIR spectroscopy with a quantum cascade laser microscope.^20,21^ Surface roughness of MPs and NPs was characterized by using a KEYENCE VK-X200 3D laser microscope (KEYENCE Corporation, Itasca, IL, USA). Measured concentrations of MPs and NPs were reported as the number of particles per liter.

The environmental conditions at each site were characterized based on total MP concentrations and eight physicochemical factors: dissolved oxygen, salinity, pH, chemical oxygen demand (COD), total phosphorus (TP), nitrate, ammonia-nitrogen, and sulfate. Details are summarized in Table S1.

Genomic DNA was extracted from 45 samples—15 each for MP, NP, and RW microbiomes, collected across 15 sites (i.e., one sample of each microbiome type per site) and subsequently sequenced. The raw sequence data have been deposited in the NCBI Sequence Read Archive (BioProject accession number PRJNA1159715). Raw sequencing data were prepared for downstream bioinformatic analysis (Text S2). Briefly, high-quality reads were obtained through quality control, followed by taxonomic classification and contig assembly. Open reading frames were then predicted for functional annotation. The resulting species classifications and functional annotations were used in the diversity and indicator analyses of MP, NP, and RW microbiomes.

Life-history strategies were inferred from functional indicators using the Y-A-S framework, as previously described.^22^ To explore the ecological roles of the three microbiomes, biochemical cycling-related genes, plastic degradation enzymes (PDEs), antibiotic resistance genes (ARGs), and virulence factors (VFs) were annotated from open reading frames. Detailed methods are shown in Text S3. Representative metagenome-assembled genomes (rMAGs) were reconstructed from all samples and dereplicated at the subspecies level (average nucleotide identity >99%) to identify potential microbial sharing events. rMAGs with high similarity between MPs or NPs and RW at the same site or river were considered indicators of sharing. Taxonomic and functional annotations were performed for both shared and nonshared rMAGs, and HGT events among these rMAGs were predicted. Detailed methods are provided in Text S4.

All data analyses and statistical tests were conducted using R (v.4.1.1)^23^ (see Text S5 for details). Briefly, principal component analysis (PCA) was used to compare environmental conditions across the sampling sites. Microbial taxonomic and functional compositions were visualized using pricipal coordinate analysis (PCoA). Differences in compositions between any two microbiome types were assessed by using permutational multivariate analysis of variance (PERMANOVA) and permutational analysis of multivariate dispersion (PERMDISP). Environmental factors were incorporated into PCoA via multiple regression to examine their correlations with the microbial composition. Mantel tests examined associations between MP taxonomic composition and MP characteristics (type, shape, and size). Pearson’s correlation coefficients assessed the relationship between taxonomic and functional dissimilarities within each microbiome type (*r* = 1 and *r* = 0 indicating complete functional dependency and redundancy, respectively). Two-sided Wilcoxon tests compared α diversity, functional abundances, and microbial sharing across microbiome types. Linear regression tested associations between total MP concentration and both functional abundances and the number of shared rMAGs, while partial Pearson correlation assessed direct associations after controlling for other environmental factors. Procrustes analysis was performed to assess the association between the taxonomic compositions of rMAGs and short reads. The significance of all tests was determined using 999 permutations, with *p*-values adjusted using the false discovery rate method. Quantitative data are presented as mean ± standard deviations.

## Results

### Characteristics of MPs and NPs and Environmental Conditions in Urban Rivers

Four polymer types of MPs (polypropylene (PP), polyethylene (PE), polystyrene (PS), and polyethylene terephthalate (PET)) were detected across all sites (total concentration range: 0.6 to 8.1 particles/L; mean: 2.5 ± 1.9 particles/L) (Table S1). These levels are comparable to those reported in other major river systems, including the Thames in the United Kingdom (22–510 particles/L),^24^ the Seine in France (0.014–4.7 particles/L),^25^ and the Yangtze in China (3.1 ± 0.3 particles/L).^26^ However, cross-study comparisons should be interpreted with caution due to methodological differences.^27^ The total NP concentrations were substantially higher (approximately 5–20 times) than MP concentrations (Table S1), consistent with previous findings,^13^ and were primarily comprised of plant remnants, mosses, and sand, ranging in size from 200 to 1000 μm (Figures S3 and S4). For MPs, consistent with observations in other urban rivers,^26,28^ the predominant polymer type, shape, and size at the majority of sites were PP, fragment, and 100–200 μm, respectively (Figure S5). Surface analysis detected plastic additives in all four polymer types, with stronger signals for phthalates and slip agents than for antioxidants and hindered amine light stabilizers (Figure S6). Signs of environmental aging were limited in PP and PE, but PET and PS showed potential hydrolytic degradation and oxidation, respectively (Figure S6). MPs also exhibited significantly greater surface roughness than NPs (Wilcoxon test, all *P* < 0.05) (Figure S7). These findings highlight the distinct physicochemical properties of MPs compared to those of NPs.

PCA of the total MP concentration and eight other environmental factors showed no significant differences in environmental conditions between rivers (PERMANOVA, *R* ^2^ = 0.541, *P* = 0.754; PERMDISP, *P* = 0.573) (Figure S8). The total MP concentrations and several anthropogenic pollution-related factors (i.e., COD, TP, and nitrate concentrations) exhibited high loadings (0.387–0.494) on the first principal components, indicating that anthropogenic disturbances strongly influence the environmental conditions of urban rivers.

### Similar Taxonomic Compositions but Distinct Indicator Species in MP and NP Microbiomes

To elucidate microbial heterogeneity among MPs, NPs, and RW, we analyzed the taxonomic compositions of the associated microbiomes were analyzed. Overall, 7756 species across 59 phyla were identified, accounting for 49.4 ± 16.5% of high-quality reads. Species classification suggested that MP and NP microbiomes were similar and both were distinct from RW microbiomes. Regarding α-diversity, MP and NP microbiomes had comparable species richness (Wilcoxon test, *P* = 0.548), which was lower than that of RW microbiomes (Wilcoxon test, all *P* < 0.001) (Figure 1a). Shannon’s diversity index did not differ significantly among the three microbiome types (Wilcoxon test, all *P* > 0.05) (Figure 1b). Regarding β-diversity, PCoA showed that MP and NP microbiomes were similar (PERMANOVA, *R* ^2^ = 0.025, *P* = 0.630; PERMDISP, *P* = 0.533), while both were significantly distinct from RW microbiomes (pairwise PERMANOVA, all *R* ^2^ > 0.178, *P* < 0.01; pairwise PERMDISP, all *P* > 0.05) (Figure 1c). The compositions of all three microbiome types were significantly influenced by the COD concentration (multiple regression, all *R* ^2^ > 0.412, *P* < 0.05) (Figure 1d). The compositions of MP microbiomes varied significantly with total MP concentration (multiple regression, *R* ^2^ = 0.656, *P* = 0.008) (Figure 1d) but not with the relative compositions of different MP types, shapes, or sizes (Mantel test, *r* = −0.019 to −0.09, all *P* > 0.05). Subsequent analyses focused on microbial variations relative to the total MP concentration irrespective of specific properties.

**Figure 1:**
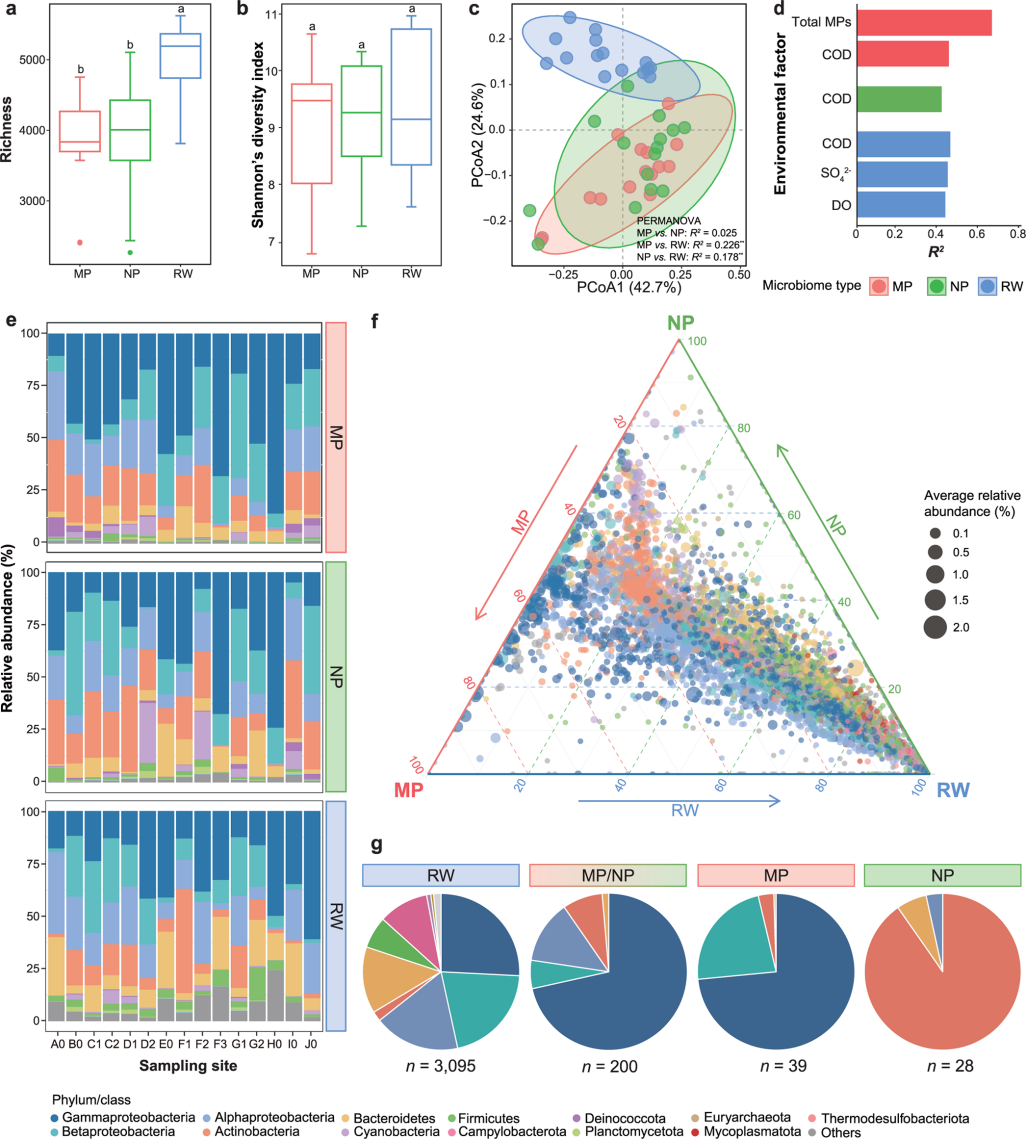
Variations in the taxonomic compositions of MP, NP, and RW microbiomes. (a) Species richness and (b) Shannon’s diversity indexes of the three types of microbiomes. The letters indicate significant differences (*P* < 0.05) between microbiomes based on the Wilcoxon test. Each box and whiskers indicate data inside and outside the interquartile range (excluding outliers), respectively. (c) Principal coordinate analysis (PCoA) ordination of the taxonomic compositions of the three types of microbiomes based on Bray–Curtis dissimilarity (**P < 0.001). Samples are colored according to the microbiome type. The ellipses represent the 90% confidence ellipse based on a multivariate t-distribution. (d) Environmental factors found to be significantly correlated with the taxonomic compositions of different types of microbiomes based on multiple linear regression (*P* < 0.05). Bars are colored according to the microbiome type. COD: chemical oxygen demand; DO: dissolved oxygen; SO_4_^2–^: sulfate. (e) Relative abundances of the classified high-quality reads at the phylum level in each sample (class level is shown for Proteobacteria). (f) Relative distributions of all of the classified species among the three types of microbiomes based on the relative abundance in each type of microbiome. The average relative abundances of species in all samples are indicated by the symbol sizes. (g) Average relative compositions of unique and shared taxonomic indicators for different types of microbiomes at the phylum level (class level is shown for Proteobacteria) based on the relative abundances in all samples. The same color scheme is used to indicate phyla or classes in panels (e–g).

The three microbiome types had similar dominant phyla, namely Proteobacteria (63.8 ± 15.3% of the total relative abundances of classified species in all samples), Actinobacteria (16.3 ± 10.9%), and Bacteroidetes (9.5 ± 7.6%) (Figure 1e), consistent with previous studies involving urban rivers.^4,29^ The relative proportions of individual species varied across the three microbiome types, especially when comparing MPs and NPs with the RW (Figure 1f). RW microbiomes had the highest number of taxonomic indicators (*n* = 3095; 54.2 ± 10.1%), while the number of shared taxonomic indicators between MP and NP microbiomes (*n* = 200; 20.8 ± 12.3%) was higher than the respective numbers of unique taxonomic indicators for MPs (*n* = 39; 5.5 ± 2.9%) and NPs (*n* = 28; 2.3 ± 1.0%) (Figure 1g and Table S3). RW microbiomes were characterized by unique taxonomic indicators from Bacteroidetes, Firmicutes, and Campylobacterota, which are free-living and commonly found in aquatic environments.^30^ Both MP and NP microbiomes included many indicators of biofilm-forming and organic pollutant-degrading capabilities (e.g., pesticides and aromatic compounds) among genera in Gammaproteobacteria (e.g., *Pseudomonas* ^31^ and *Janthinobacterium* ^32^). Despite similarities in taxonomic compositions and shared indicators between MP and NP microbiomes, the former featured unique taxonomic indicators from Gammaproteobacteria (Figure 1g and Table S3), including strains previously reported to be involved in plastic degradation (e.g., ,^33^ ,^34^ and^35^) and denitrification (e.g.,^36^ and^37^). In contrast, unique NP taxonomic indicators were predominantly associated with Actinobacteria and known to degrade various hydrocarbons (e.g.,^38^ and *Nocardioides* sp. CF8 and sp. JQ2195 ^39^). In summary, specialized species preferentially colonize MPs versus NPs.

### Unique MP Microbiome Life-history Strategies despite Similarities in Functional Composition to NP Microbiomes

Similarities also were observed in functional diversity (Wilcoxon test, all *P* > 0.05) (Figure 2a,b) and composition (PERMANOVA, *R* ^2^ = 0.042, *P* = 0.227; PERMDISP, *P* = 0.096) (Figure 2c) between MP and NP microbiomes, and both significantly differed from RW microbiomes (functional richness: Wilcoxon test, all *P* < 0.001, Figure 2a; composition: pairwise PERMANOVA, all *R* ^2^ > 0.109, *P* < 0.01 and pairwise PERMDISP, all *P* > 0.05, Figure 2c). Variations in the functional compositions of MP microbiomes were significantly associated with the total MP concentration (multiple regression, *R* ^2^ = 0.489, *P* = 0.036) (Figure 2d). MP microbiomes exhibited functional dependency, indicated by strong correlations between functional and taxonomic compositions (Pearson’s *r* = 0.691, *P* < 0.001), whereas NP and RW microbiomes showed functional redundancy (Pearson’s *r* = 0.239–0.336, all *P* < 0.05) (Figure S9).

**Figure 2:**
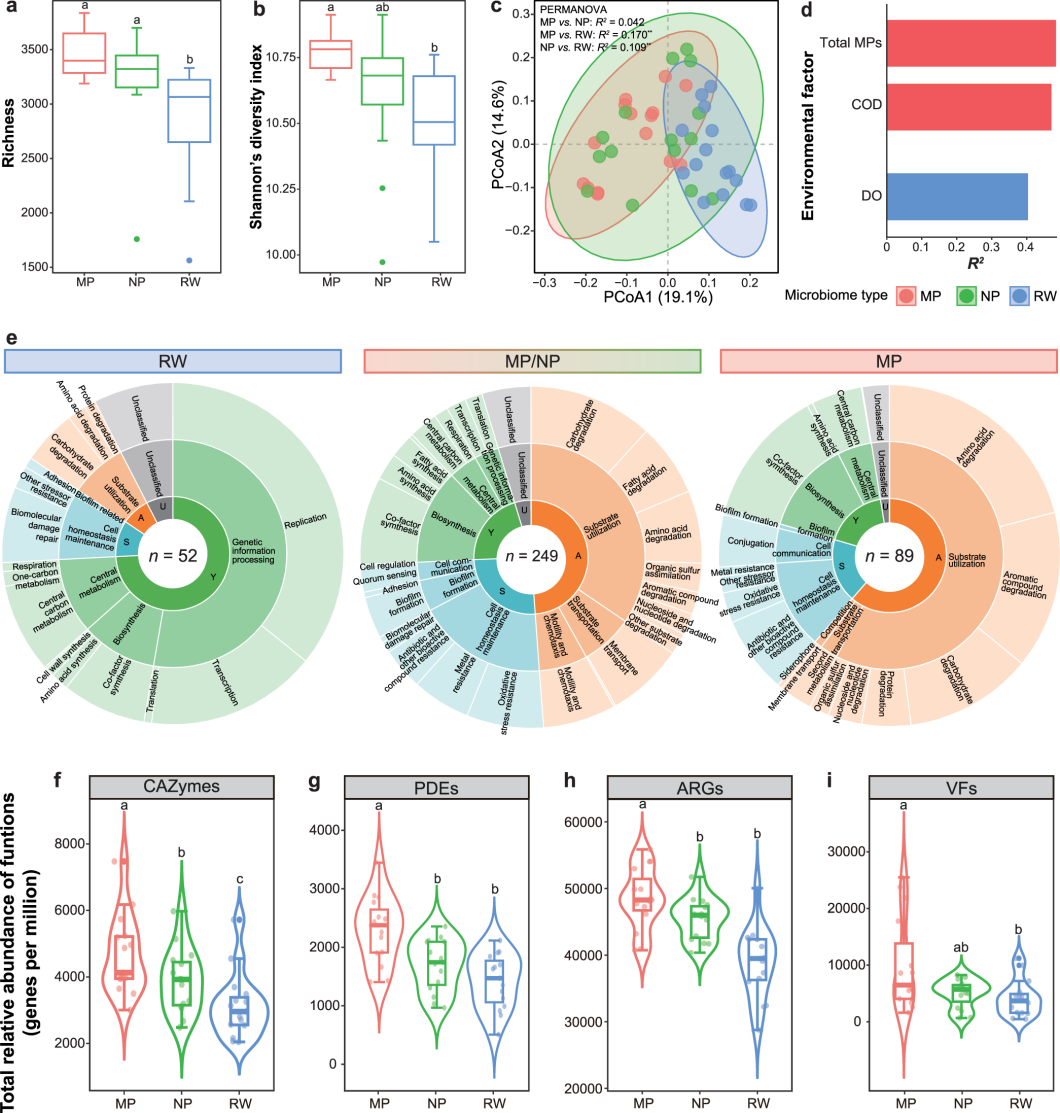
Variations in the functional compositions and ecological roles of MP, NP, and RW microbiomes. (a) Functional richness and (b) Shannon’s diversity indexes of the three types of microbiomes. (c) Principal coordinate analysis (PCoA) ordination of the functional compositions of the three types of microbiomes based on the Bray–Curtis dissimilarity (**P < 0.001). Samples are colored according to the microbiome type. The ellipses represent the 90% confidence ellipse based on a multivariate t-distribution. (d) Environmental factors found to be significantly correlated with the functional compositions of different types of microbiomes based on multiple linear regression (*P* < 0.05). Bars are colored according to the microbiome type. COD: chemical oxygen demand; DO: dissolved oxygen. (e) Life-history strategies of different types of microbiomes inferred from unique and shared functional indicators. From inside to outside, the rings indicate the proportions of the Y-A-S strategies and their subcategories based on the average relative abundances in all samples. (f–i) Total relative abundances of (f) carbohydrate-active enzymes (CAZymes), (g) putative plastic degradation enzymes (PDEs), (h) antibiotic resistance genes (ARGs), and (i) virulence factors (VFs) in MP, NP, and RW microbiomes. The letters indicate significant differences (*P* < 0.05) between microbiomes according to the Wilcoxon test. Each box and whiskers indicate data inside and outside the interquartile range (excluding outliers), respectively.

To understand how microbes adapted to their habitats, functional indicators related to microbial life-history strategies were compared among the three microbiome types by Y-A-S strategy classification^22^ (Figure 2e and Table S4). MP and NP microbiomes had a higher number of shared functional indicators (*n* = 249; 11.0 ± 7.3% of relative abundance of annotated functions in all samples) than the numbers of functional indicators unique to MP (*n* = 89; 2.4 ± 1.2%) and RW (*n* = 52; 1.23 ± 1.1%) microbiomes. No unique functional indicators were found for the NP microbiomes. Many functional indicators in RW microbiomes were associated with the Y-strategy, predominantly the genetic information processing subcategory. In contrast, functional indicators shared between MP and NP microbiomes were mainly associated with the A-strategy, including functions related to the utilization and transportation of various substrates (e.g., carbohydrates, amino acids, and derivatives, and fatty acids), motility, chemotaxis, and competition, and the S-strategy, including cell homeostasis maintenance, biofilm formation, and cell communication.

MP microbiomes could be distinguished from NP and RW microbiomes by unique functional indicators associated mainly with A- and S-strategies (Figure 2e and Table S4). Within the A-strategy subcategory of aromatic compound degradation, benzoate degradation comprised a large proportion of unique functional indicators in MP microbiomes. The S-strategy subcategories of cell homeostasis maintenance and cell communication were predominant among the unique functional indicators, which were mainly associated with resistance to antibiotics and other bioactive compounds and their conjugation.

### Shared and Unique Ecological Roles among MP, NP, and RW Microbiomes

Microbiomes’ taxonomic and functional compositions can influence their ecological roles in ecosystems.^40^ Studies have focused on three MP microbiome functional categories related closely to riverine ecosystem services and health: biochemical cycling, plastic degradation, and antibiotic resistance and virulence.^9,29,41^ A comparative analysis of these three categories was performed to elucidate similarities and differences in the ecological roles of the MP, NP, and RW microbiomes.

#### Biochemical Cycling

MP and NP microbiomes had similar biochemical cycling-related functional compositions (PERMANOVA, *R* ^2^ = 0.033, *P* = 0.352; PERMDISP, *P* = 0.103), and both significantly differed from RW microbiomes (pairwise PERMANOVA, all *R* ^2^ > 0.225, *P* < 0.01; pairwise PERMDISP, all *P* > 0.05) (Figure S10a). Although associated metabolic processes were largely present across the three microbiomes, the relative abundances of the specific processes varied. RW microbiomes had significantly higher relative abundances (Wilcoxon test, all *P* < 0.05) of processes such as carbon fixation (Figure S10e), nitrate reduction (Figure S10f), and sulfide oxidation (Figure S10g) than MP and NP microbiomes. Compared with RW microbiomes, MP and NP microbiomes had a significantly higher relative abundance of carbohydrate-active enzymes (CAZymes) (Wilcoxon test, all *P* < 0.05) (Figure 2f), consistent with their significantly higher relative abundances of organic carbon oxidation (Wilcoxon test, all *P* < 0.05) (Figure S10e).

Despite the similar metabolic profiles of MP and NP microbiomes, the former had higher relative abundances of nitrate and nitric oxide reduction-related functions (Wilcoxon test, all *P* < 0.05) (Figure S10f). Moreover, MP microbiomes had the highest relative abundance of CAZymes involved in complex carbohydrate degradation (e.g., alginate-oligosaccharide, mannooligosaccharide) (Wilcoxon test, all *P* < 0.05) (Figure S11), and the total relative abundance of CAZymes was positively correlated with the total MP concentration (linear regression, *R* ^2^ = 0.480, *P* = 0.003; partial Pearson’s *r* = 0.625, *P* = 0.033) (Figure S12a).

#### Plastic Degradation

MP microbiomes exhibited a plastic degradation potential distinct from NP and RW microbiomes, demonstrated by their distinct compositions of putative PDEs (pairwise PERMANOVA, all *R* ^2^ > 0.167, all *P* < 0.05; pairwise PERMDISP, all *P* > 0.05) (Figure S10b) and highest total relative abundance of putative PDEs (Wilcoxon test, all *P* < 0.005) (Figure 2g). Moreover, putative PDE abundance in MP microbiomes was positively correlated with the total MP concentration (linear regression, *R* ^2^ = 0.360, *P* = 0.011; partial Pearson’s *r* = 0.514, *P* = 0.045) (Figure S12b).

Among the 43 identified putative PDEs, phenylacetaldehyde dehydrogenase, involved in PS degradation,^42^ was most abundant in all three microbiome types, particularly MP microbiomes (Wilcoxon test, all *P* < 0.01) (Figure S10h). Other prevalent putative PDEs in all three microbiome types, including catalase, xylanase, and esterase (Figure S10h), decompose and depolymerize various types of plastics,^43^ including PE, PS, and PET, which were detected at all sampling sites.

#### Antibiotic Resistance and Virulence

Compared with NP and RW microbiomes, MP microbiomes had significantly different compositions of ARGs (pairwise PERMANOVA, all *R* ^2^ > 0.076, *P* < 0.05; pairwise PERMDISP, all *P* > 0.05) (Figure S10c) and VFs (pairwise PERMANOVA, all *R* ^2^ > 0.051, *P* < 0.05; pairwise PERMDISP, all *P* > 0.05) (Figure S10d). MP microbiomes also had the highest total relative abundance of ARGs (Wilcoxon test, all *P* < 0.005) (Figure 2h), and a higher total relative abundance of VFs than RW microbiomes (Wilcoxon test, *P* = 0.039) (Figure 2i), and the total relative abundances of ARGs (linear regression, *R* ^2^ = 0.265, *P* = 0.029; partial Pearson’s *r* = 919, *P* = 0.003) (Figure S12c) and VFs (linear regression, *R* ^2^ = 0.603, *P* < 0.001; partial Pearson’s *r* = 0.789, *P* = 0.013) (Figure S12d) were positively correlated with the total MP concentration.

Among the 120 identified ARGs, multiple drug resistance genes were the most predominant subtype in all three microbiome types and significantly most abundant in MP microbiomes (Wilcoxon test, all *P* < 0.05) (Figure S10i). Of the 3779 identified VFs, adherence was the predominant category across all three microbiome types, while nutritional/metabolic factors and biofilm-related VFs were more abundant in both MP and NP microbiomes than in RW microbiomes (Wilcoxon test, all *P* < 0.01) (Figure S10j).

### Differential Microbial Sharing Capabilities between MPs and NPs with RW

The microbial sharing capabilities of MPs and NPs with the RW were compared. rMAGs were used to investigate the influence of MPs and NPs on overall microbial partitioning in riverine ecosystems. Comparison of rMAG taxonomic profiles with short reads confirmed that the recovered rMAGs captured the most dominant phyla or classes (Figure S13a) and were highly correlated with short-read profiles (Procrustes test, *M* ^2^ = 0.279, *P* < 0.001) (Figure S13b), indicating their representativeness. Among the reconstructed rMAGs, 234 were shared and nonshared rMAGs, accounting for 22.8 ± 11.2% of total reads in all samples (Table S5). Sharing events were observed between MPs or NPs and the RW from the same sampling site and along the same river (Figure 3a,b). As expected, greater numbers of rMAGs were shared between the two types of particles and RW from the same sites or rivers than between samples from geographically separated sites (Wilcoxon test, all *P* < 0.05) (Figure 3c,d). Although the numbers of shared rMAGs between MPs or NPs and RW from the same sites or rivers were similar (*n* = 11 ± 9 vs 8 ± 7; Wilcoxon test, *P* = 0.246), the number of shared rMAGs between MPs and RW and the total MP concentration were significantly and positively correlated (linear regression, *R* ^2^ = 0.357, *P* = 0.011; partial Pearson’s *r* = 0.649, *P* = 0.039) (Figure S14).

**Figure 3:**
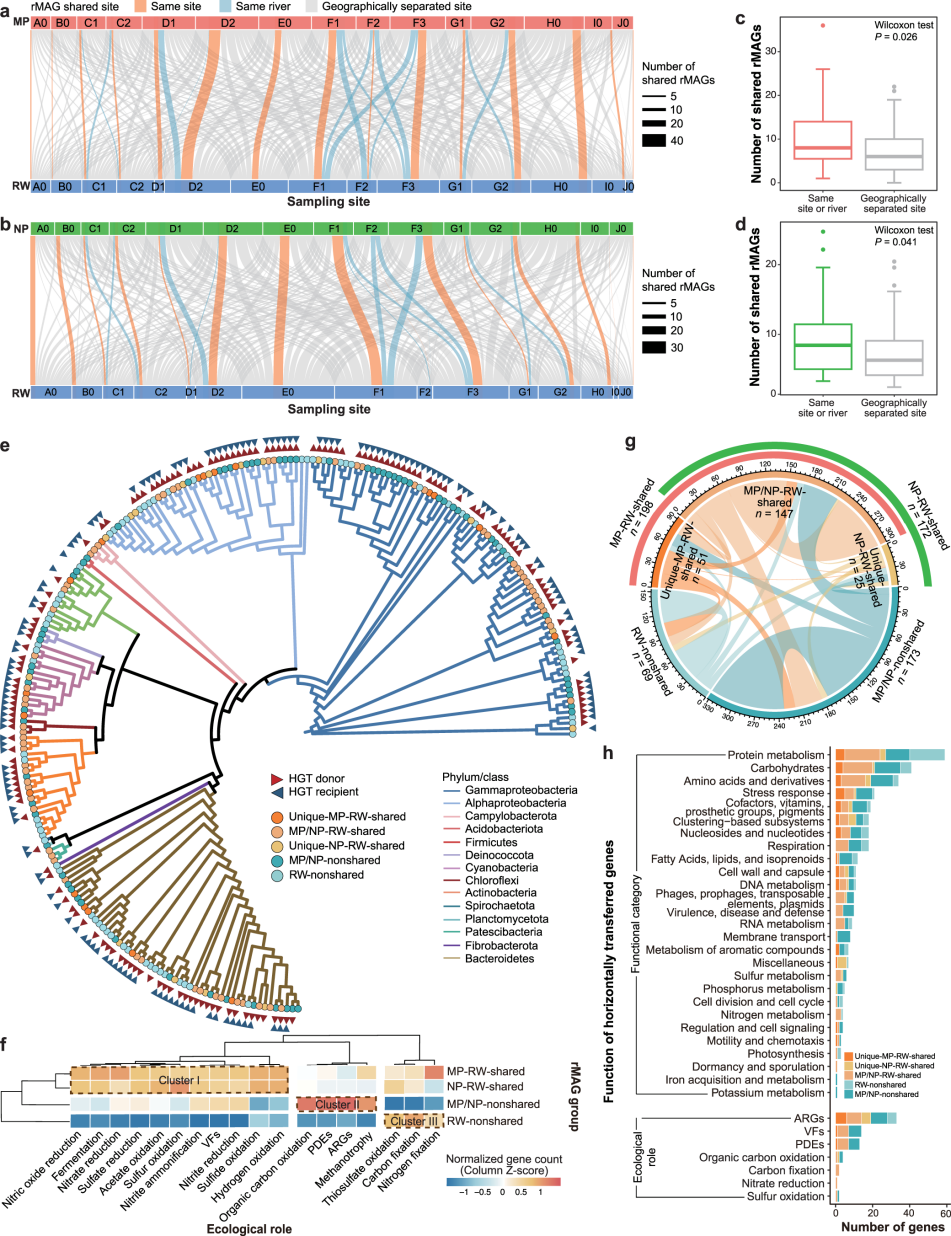
Microbial sharing and horizontal gene transfers (HGTs) in MP, NP, and RW microbiomes. (a, b) Shared representative metagenome-assembled genomes (rMAGs) (a) between MPs and RW and (b) between NPs and RW. The width of each band connecting two environments corresponds to the number of shared rMAGs, and the color indicates the site where sharing occurred. (c, d) Number of shared highly similar rMAGs (c) between MPs and RW and (d) between NPs and RW from samples collected along the same rivers or from geographically separated sites. Each box and whiskers indicate data inside and outside the interquartile range (excluding outliers), respectively. (e) Maximum likelihood phylogenic tree of shared and nonshared rMAGs at the phylum level (class level is shown for Proteobacteria). The branch color corresponds to the phylum or class, and the node color indicates the sharing status of each rMAG. The triangles outside the nodes represent the roles of rMAGs in HGT and are colored to indicate whether they are donors or recipients. (f) Ecological roles of shared and nonshared rMAGs. A hierarchical tree was generated using Ward’s method and Euclidean distance. (g) Predicted HGT events among shared and nonshared rMAGs. The bands connect donors and recipients; the width of each band corresponds to the number of HGTs, and the color indicates the donor. (h) Functional categories based on the SEED database and ecological roles of horizontally transferred genes among shared and nonshared rMAGs.

The 234 rMAGs were categorized by habitat and potential for sharing (Table S5). Ninety-seven rMAGs showed evidence of sharing, with a slightly higher number shared between MPs and RW (i.e., MP–RW-shared, *n* = 81) than between NPs and RW (i.e., NP–RW-shared, *n* = 79). Eighteen rMAGs were exclusively MP–RW-shared (i.e., unique-MP–RW-shared), while 16 were exclusively NP–RW-shared (i.e., unique-NP–RW-shared). The remaining 63 rMAGs were shared between either MPs or NPs and RW (i.e., MP/NP–RW-shared). Of the 137 rMAGs lacking evidence of sharing, 68 were exclusively detected in RW (i.e., RW-nonshared), and 69 were present in both MPs and NPs (i.e., MP/NP-nonshared), indicating specificity for a free-living or particle-attached lifestyle.

Taxonomic analysis showed differences in composition between shared and nonshared rMAGs (Figure 3e and Table S5). Although both groups were dominated by Gammaproteobacteria and Alphaproteobacteria, shared rMAGs belonged to a broader range of phyla, including Campylobacterota and Patescibacteria, which were absent from nonshared rMAGs. Notably, shared rMAGs included putative pathogens from Campylobacteria, such as *Aliarcobacter* spp.,^44^ suggesting that microbial sharing may introduce concerning taxa into riverine ecosystems. The taxonomic compositions of the MP-RW-shared and NP–RW-shared rMAGs were similar. Compared with shared rMAGs, MP/NP-nonshared rMAGs had a higher proportion of Actinobacteria, while RW-nonshared rMAGs had higher proportions of Bacteroidetes and Firmicutes. Functional analysis showed variations in ecological roles between shared and nonshared rMAGs (Figure 3f). MP–RW-shared and NP–RW-shared rMAGs carried greater total numbers of genes encoding VFs and nitrogen- and sulfur cycling-related functions than nonshared rMAGs (cluster I in Figure 3f). Specifically, MP–RW-shared rMAGs harbored more genes related to nitrogen processes (e.g., nitrogen fixation, nitrate reduction, and nitric oxide reduction) than NP–RW-shared rMAGs. Compared with shared rMAGs, MP/NP-nonshared rMAGs carried more genes related to organic carbon oxidation, methanotrophy, putative PDEs, and ARGs (cluster II in Figure 3f), whereas RW-nonshared rMAGs carried more genes related to carbon fixation and thiosulfate oxidation (cluster III in Figure 3f).

### Shared rMAGs Drive HGT Events in Microbiomes

Microbial sharing can influence the functions of receiving microbiomes by providing exogenous genetic material for HGT.^17,45^ The numbers and types of genes transferred through HGT events were predicted to investigate potential gene exchanges between shared and nonshared rMAGs in the three microbiome types. The 465 detected HGT events involved 183 of the 234 shared and nonshared rMAGs; of these, 128 rMAGs acted as both HGT donors and recipients (Figure 3e and Table S5). MP–RW-shared rMAGs were the primary donors, contributing the highest number of transferred genes (*n* = 198), followed by MP/NP-nonshared (*n* = 173) and NP–RW-shared (*n* = 172) rMAGs; RW-nonshared rMAGs (*n* = 69) contributed the fewest HGT events (Figure 3g). Notably, shared rMAGs contributed nearly half of all genes received by nonshared rMAGs, with MP–RW-shared rMAGs (*n* = 52) transferring more genes than NP–RW-shared rMAGs (*n* = 45) (Figure 3g). Genes transferred between shared and nonshared rMAGs were mainly related to metabolism (e.g., proteins [*n* = 60], carbohydrates [*n* = 42], amino acids and derivatives [*n* = 34]), and stress responses (*n* = 22) (Figure 3h). ARGs were more frequently transferred (*n* = 33) than genes related to biochemical cycling (*n* = 9), VFs (*n* = 9), and putative PDEs (*n* = 8) (Figures 3h and S15) and were the only functional category transferred among all shared and nonshared rMAGs (Figure S15a).

## Discussion

The plastisphere, a novel feature of the Anthropocene,^18^ is an influential component of vulnerable urban river ecosystems; however, its ecological impacts remain unclear. While our understanding of the plastisphere has grown,^10^ we lack comprehensive knowledge of the inhabiting microbes and their ecological functions and interactions with riverine ecosystems, particularly in comparison with microbes associated with NPs. An understanding of these plastisphere traits is crucial for elucidating microbial partitioning and functional shifts in MP-affected riverine ecosystems. Accordingly, in this study, we collected microbiomes associated with MPs, NPs, and RW from 10 urban rivers in Hong Kong, which are characterized by dynamic flow and varying levels of MP pollution. Metagenomic analysis were performed to comprehensively compare the taxonomic and functional profiles of the three microbiome types, encompassing the diverse range of microbial activity, including those with low metabolic rates, slow growth, or dormancy.^46,47^ MP and NP microbiomes were found to share taxonomic and functional similarities; however, MP microbiomes were uniquely associated with specific taxa and life-history strategies, which potentially contributed to their unique ecological roles. Furthermore, MP microbiomes exhibited unique shared microbes and HGT capabilities and may have influenced the surrounding RW microbiomes differently from NP microbiomes.

MP and NP microbiomes from the 10 urban rivers exhibited relatively similar taxonomic and functional compositions, suggesting that both anthropogenic and natural particles support the establishment of microbial assemblages with comparable traits (i.e., typical biofilm-related colonizers and functions), regardless of their inherent properties. This similarity can be attributed to the particle types’ similar roles as resource-rich, yet stressed, niches.^48^ Although generally less bioavailable than NPs, MPs readily absorb nonpolar organic matter^49^ and provide comparable amounts of carbons and nutrients for microbial utilization and growth. Elevated COD and TP, likely from anthropogenic sources, were observed at many sites and may have been sorbed by extracellular polymeric substances in MP and NP biofilms.^50^ Thus, both types of particle-attached microbiomes feature pollutant-degrading taxa and functions associated with the utilization of diverse substrates. Overall, MP and NP microbiomes tended to exhibit similar adaptations to particle attachment.

Despite these similarities, however, MP microbiomes exhibited distinct taxonomic and functional traits and high functional dependency with minimal overlap in functions among species. This appears to be a crucial adaptation to enable complex organic matter degradation, a multistep process requiring diverse taxa with specialized functions.^51^ Accordingly, MP microbiomes were enriched in CAZymes potentially involved in complex carbohydrate degradation, likely due to the higher hydrophobicity of MPs compared to NPs (e.g., mineral and woody particles), which enhances their affinity for complex organic compounds.^52^ MP microbiomes also exhibited substantial potential for plastic degradation, as evidenced by the enrichment of putative PDEs and potential plastic degraders from the genus *Pseudomonas*. MP microbiomes were also closely associated with the degradation of benzoates, which are common components in certain plasticizers.^53^ These results highlight the potential influence of MPs’ unique physicochemical properties on the composition and function of associated microbiomes. Furthermore, MP microbiomes showed increased potential for nitrate and nitric oxide reduction compared with NP microbiomes, suggesting a potentially important role for the former in nitrogen cycling. The denitrification potential observed in MP microbiomes might contribute to the degradation of plastics^54^ and carbohydrates.^55,56^ Additionally, MP microbiomes displayed an increased potential for antibiotic resistance, possibly due to exposure to toxic additives (e.g., phthalates) and the ability of MPs to accumulate toxic substances (e.g., antibiotics and endocrine disruptors), likely facilitated by their greater surface roughness,^57^ which can select and enrich ARGs within associated microbiomes. MP microbiomes also were associated with conjugation, which can further amplify antibiotic resistance by facilitating the transfer of ARGs between microbes.^58^

RW microbiomes were found to diverge from MP and NP microbiomes, exhibiting increased potential for diverse biochemical cycling processes such as carbon fixation and thiosulfate oxidation, which are metabolic processes suited to resource-limited RW environments.^59^ Over time, however, these traits of RW microbiomes might be influenced through interactions with MP and NP microbiomes. Microbial sharing between particle-attached and free-living microbiomes was evident not only within sampling sites but also along the lengths of rivers, suggesting that both MPs and NPs may transport microbes over long distances. Moreover, a substantial number of shared rMAGs overlapped between MPs and NPs, likely due to their similar microbial assemblages. As a result, microbes shared between NPs and RW could also colonize MPs and be shared with RW.^15^ Alongside the microbial sharing common to both MPs and NPs, distinct rMAGs with different functional capabilities, despite similar taxonomies, were shared exclusively between MPs and RW, as well as between NPs and RW. Compared with the unique-NP–RW-shared rMAGs, the unique-MP–RW-shared rMAGs contained more genes associated with nitrate and nitric oxide reduction, which may influence denitrification dynamics between particle-attached and free-living microbiomes.^60^

Microbial sharing not only introduce individual microbes into receiving microbiomes but also can facilitate subsequent HGTs between different taxa within those microbiomes.^45^ Indeed, HGT was observed widely between shared and nonshared rMAGs across the three microbiome types, indicating the potential risk of functional alterations.^17^ Notably, unique-MP–RW-shared rMAGs contributed more to HGT events than unique-NP–RW-shared rMAGs. This enhanced HGT capacity of unique-MP–RW-shared rMAGs may be important for their successful colonization of and coexistence within MP and RW microbiomes.^61^ The unique physicochemical properties of MPs may increase the selective pressure on associated microbes, possibly explaining why the leading transferred genes were mainly related to metabolism and stress responses: these genes can promote resistance and maintain growth within MP microbiomes. Furthermore, unique-MP–RW-shared rMAGs transferred a higher proportion of genes to RW-nonshared rMAGs than unique-NP–RW-shared rMAGs; accordingly, the former group might alter the metabolic capabilities and ecological roles of RW microbiomes.^62^

While this study provides a comprehensive comparative evaluation of MP and NP microbiomes, it has a few limitations. First, the samples were collected from only 15 sites; although these sites are representative of major urban river systems susceptible to anthropogenic influences, the samples may not have captured the full variability of these ecosystems. Moreover, MPs and NPs with different properties (e.g., type and size) were pooled, respectively, rather than analyzed according to their specific physical and chemical characteristics, which may have masked particle-specific effects on associated microbiomes. Future studies that expand sampling to more sites along the lengths of rivers and wider geographical regions, while controlling for particle properties, could verify the observed differences in microbial assemblages between MPs and NPs and provide deeper insights into spatial variability. Second, although metagenomics analysis revealed taxonomic and functional potentials, it did not capture cellular metabolic activity or gene expression. Putative functions linked to ecological roles, such as plastic degradation, remain poorly understood because they depend on complex environmental conditions beyond the gene presence. Moreover, correlations derived from metagenomic data indicate associations rather than causation. Follow-up laboratory experiments, including chemical quantification (e.g., MP degradation products) and antimicrobial susceptibility tests,^63^ along with the integration of metatranscriptomics and metabolomics,^64^ are needed to gain a more comprehensive understanding of microbial activities and ecological roles. Third, microbial sharing analysis relied on identifying highly similar reconstructed rMAGs, which represented only a relatively small proportion of the total metagenomic data, and the viability and activity of the shared microbes was unclear. A more effective approach to confirm the extent of microbial sharing and the involved taxa might involve tracking the presence of different fluorescently labeled microbial markers in the three microbiome types^65^ during incubation experiments involving MPs and NPs. Viability and activity could also be assessed using flow cytometry with fluorescence labeling.^66^ Additionally, variations in the completeness of reconstructed rMAGs may have affected the functional comparisons^67^ and HGT predictions.^68^ In future studies, deep sequencing with both short and long reads could generate higher-quality rMAGs and enable more accurate genome comparisons.^69^

## Environmental Implications

The uniqueness of the plastisphere associated with MPs should be a crucial consideration when assessing their ecological impact on aquatic environments.^48^ Using major urban rivers with varying MP pollution levels as a model, this study demonstrates that the introduction of MPs expands the ecological niches of particle-attached microbes, highlighting how microbial lifestyles (particle-attached vs free-living) more strongly influence aquatic microbial assemblages than particle types (natural vs anthropogenic). Although the MP microbiomes shared similarities with the NP microbiomes, they also played distinct ecological roles, possibly due to selection by the distinctive properties of MPs; accordingly, MPs may uniquely impact the functions and health of aquatic ecosystems. These distinctive ecological roles include enhanced potentials for degrading complex carbohydrates and plastics, which could alter nutrient profiles in aquatic food webs, as well as physicochemical and ecotoxic properties.^10^ Furthermore, MP microbiomes harbored elevated levels of ARGs, which may pose threats to ecosystems and public health. Our results highlight the need to evaluate the uniqueness and ecological impacts of the plastisphere by comparing it with not only free-living microbiomes but also microbiomes associated with co-occurring NPs in aquatic environments. This approach is in contrast to numerous studies that have characterized MPs as a distinct niche in aquatic environments based solely on comparisons between the plastisphere and free-living microbiomes.^70,71^

The impacts of MPs extend beyond their associated microbiomes, as microbial sharing between MPs and the surrounding water, coupled with HGTs, can disperse plastisphere traits and thus alter taxonomic and functional dynamics in the broader ecosystem. Although MPs are a growing environmental concern, NPs remain dominant in aquatic environments.^13^ Consequently, the ability of NPs to harbor microbes with concerning functions (e.g., VFs and ARGs) and facilitate their transport remains an important issue. However, the increasing global production and persistence of plastics suggest a future in which the ecological influence of MPs may rival that of NPs. Furthermore, the assemblages and concerning functional potentials of MP microbiomes and the extent of sharing varied significantly with the degree of MP accumulation. This finding emphasizes the urgent need to curb MP pollution and thoroughly assess its long-term, ecosystem-wide consequences, particularly through the lens of microbiome alteration, with the aim of maintaining the overall function and health of aquatic ecosystems.

## Supplementary Material





## Notes

An overview of sampling site and analysis strategies; representative optical photothermal infrared spectra of four MP polymer types and microscopic images of different NP types; heatmaps of relative MP proportions at each sampling site based on MP properties; PCA of the nine measured environmental factors; relationships between functional and taxonomic similarity in tested microbiomes; ecological roles of microbiomes; relative abundances of functions related to typical complex carbohydrate degradation in tested microbiomes; relationships between the relative abundances of CAZymes, PDEs, ARGs, and VFs and total MP concentration in MP microbiomes; relationship between the total number of shared rMAGs between MPs and surrounding RW and total MP concentration; predicted HGTs of ARGs, VFs, and PDEs among shared and nonshared rMAGs (PDF)
Sampling site and environmental measurements in urban rivers in Hong Kong; taxonomic and functional indicators of different microbiome types; taxonomic, sharing, and HGT profiles of shared and nonshared rMAGs (XLSX)
