# Supplementary material for: Ecological Roles and Shared Microbes Differentiate the Plastisphere from Natural Particle-Associated Microbiomes in Urban Rivers
Source: Environ Sci Technol. 2025 Aug 8;59(32):17298–309. doi: 10.1021/acs.est.5c06538 (PMC12369012; doi:10.1021/acs.est.5c06538)
Supplement: Supplementary file 1 [file es5c06538_si_001.pdf]

Supplementary Information

Ecological roles and shared microbes differentiate the plastisphere from natural particle-associated microbiomes in urban rivers

Yingyu Bao,[1] Yuen-Wa Ho,[2,3] Zhiyong Shen,[1] Edmund Y. Lam[4], James K. H. Fang,[2,3]* Kenneth M. Y. Leung,[1,3,5] and Patrick K. H. Lee[1,3,6]*

[1]School of Energy and Environment, City University of Hong Kong, Hong Kong SAR 999077, China

[2]Department of Food Science and Nutrition and Research Institute for Future Food, The Hong Kong Polytechnic University, Hong Kong SAR 999077, China

[3]State Key Laboratory of Marine Pollution, City University of Hong Kong, Hong Kong SAR 999077, China

[4]Department of Electrical and Electronic Engineering, The University of Hong Kong, Hong Kong SAR 999077, China

[5]Department of Chemistry, City University of Hong Kong, Hong Kong SAR 999077, China

[6]Low-Carbon and Climate Impact Research Centre, City University of Hong Kong, Hong Kong SAR 999077, China

***Correspondence**

Patrick K. H. Lee: B5423, Yeung Kin Man Academic Building, School of Energy and Environment, City University of Hong Kong, Tat Chee Avenue, Kowloon, Hong Kong SAR, China; E-mail: patrick.kh.lee@cityu.edu.hk; Tel: (852) 3442-4625; Fax: (852) 3442-0688.

James K. H. Fang: TU313, Yip Kit Chuen Building, Department of Food Science and Nutrition, The Hong Kong Polytechnic University, Hung Hom, Kowloon, Hong Kong SAR, China; E-mail: james.fang@polyu.edu.hk; Tel: (852) 3400-8703; Fax: (852) 2364-9909.

Supplementary Information includes 35 pages, 5 text sections, 15 figures, and 5 tables.

Table of contents

Text S1. Sample collection, particle characterization, and physicochemical analyses.........................S4
Text S2. Metagenomic sequencing, bioinformatics processing, and diversity and indicator analyses S7
Text S3. Inference of life-history strategies and determination of potential ecological roles S9
Text S4. Detection of potential microbial sharing and horizontal gene transfer (HGT) events S11
Text S5. Data analysis and statistics S13

Figure S1. Overview of the sampling and analysis strategy for the study. (a) A map of the 15 sampling sites across 10 urban rivers in Hong Kong. Multiple sampling sites in the same river are labeled with numeric values increasing from upstream to downstream. (b) A schematic illustrating the workflow of the sampling process and key data analyses. S15

Figure S2. Representative optical photothermal infrared (O-PTIR) spectra of four polymer types of MPs in water samples. S17

Figure S3. Representative microscopic images of different types of NPs in water samples..............S18

Figure S4. Hierarchical clustering heatmaps showing the relative proportions of NPs at each sampling site by (a) type and (b) size...S19

Figure S5. Hierarchical clustering heatmaps showing the relative proportions of MPs at each sampling site based on (a) polymer type, (b) shape, and (c) size. S20

Figure S6. Representative optical photothermal infrared (O-PTIR) spectra showing potential plastic additives and signs of aging in four MP polymer types from water samples. S21

Figure S8. Principal component analysis (PCA) plot showing the nine environmental factors measured in the samples. S23

Figure S9. Relationships between functional and taxonomic similarity (measured as 1 − Bray–Curtis dissimilarity) in MP, NP, and RW microbiomes. S24

Figure S10. Ecological roles of MP, NP, and RW microbiomes. (a–d) Principal coordinate analysis (PCoA) ordination of functional compositions related to (a) biochemical cycling, (b) plastic degradation, (c) antibiotic resistance, and (d) virulence in the three types of microbiomes based on Bray–Curtis dissimilarity (** P < 0.001; * P < 0.05). (e–g) Biogeochemical cycling diagrams showing the major metabolic processes involved in (e) carbon, (f) nitrogen, and (g) sulfur cycling. (h–j) Heatmaps showing the relative abundances of the major (h) PDEs, (i) ARG subtypes, and (j) VF subcategories in the three types of microbiomes. S25

Figure S11. Relative abundances of functions related to the degradation of typical complex carbohydrates in MP, NP, and RW microbiomes.. ....S27

Figure S12. Relationships between the relative abundances of (a) carbohydrate-active enzymes (CAZymes), (b) plastic degradation enzymes (PDEs), (c) antibiotic resistance genes (ARGs), and (d) virulence factors (VFs) and the total MP concentration in MP microbiomes. S28

Figure S13. (a) Relative taxonomic compositions of rMAGs at the phylum level in each sample (class level for Proteobacteria) and (b) Procrustes analysis comparing the microbial taxonomic compositions of rMAGs and short reads based on Bray−Curtis dissimilarity. S29

Figure S14. Relationship between the total number of shared rMAGs between MPs and the surrounding RW and the total MP concentration. S30

Figure S15. Predicted HGTs of (a) antibiotic resistance genes (ARGs), (b) virulence factors (VFs), and (c) putative plastic degradation enzymes (PDEs) among shared and non-shared rMAGs. S31

Text S1. Sample collection, particle characterization, and physicochemical analyses

Sampling was conducted at 15 sites across 10 major urban rivers in Hong Kong in February 2023; multiple sites were sampled in four rivers (**Fig. S1a**). These rivers are intricately intertwined with Hong Kong's urban landscape. The average length and catchment area of the studied rivers were approximately 18 ± 21 km and 18 ± 13 km^2, respectively (**Table S1**). GPS coordinates for all sampling sites are provided in **Table S1**, and an overview of the sampling and analysis strategy is provided in **Fig. S1b**. At each site, two 50-L sets of surface water (top 0.2 m) were collected and sequentially filtered on-site using stainless-steel sieves with 5,000-µm and 100-µm pores. Particles retained on the 100-µm sieve were rinsed into sterile glass bottles using filtered RW. One set was used for MP and NP microbiome analysis and another set for MP and NP quantification. Additionally, 2-L aliquots of filtered water were collected in sterile sample bags (Bkmam, Changde, China). To monitor potential airborne MP contamination and ensure data reliability, field blanks—open, pre-cleaned Petri dishes—were placed near the sampling area. No suspected MP particles (100–5,000 µm) were observed under stereomicroscopy (SMZ1270i; Nikon, Tokyo, Japan) (**Table S2**), indicating negligible airborne contamination. In the laboratory, 0.5–1 L of collected water from each site was immediately filtered using sterile 0.2-µm polyethersulfone filters (Supor 200; Gelman, Ann Arbor, MI, USA) to concentrate microbes for RW microbiome analysis. The remaining water samples were reserved for physicochemical analyses. All samples were stored at −80°C until further processed.

To obtain MPs and NPs for microbiome analysis, the particle types in water samples were initially differentiated through visual sorting by color and morphology under a stereomicroscope[1]. The composition of each subset of identified particles in each water sample was confirmed using optical photothermal infrared (O-PTIR) spectroscopy (mIRage IR microscope; Photothermal Spectroscopy Corp., Santa Barbara, CA, USA)[2]. Particles were

illuminated with a mid-IR pulsed tunable laser, using a 532 nm detection laser with a 10-second exposure time. The O-PTIR spectra of the particles were then acquired in the wavenumber range of 200 to 4170 cm^{-1} using the PTIR Studio 4.4 software. These spectra were compared against the polymeric compounds library (KnowItAll Informatics System 2021) to classify MPs, with any unmatched particles categorized as NPs. The acquired O-PTIR spectra of the identified MPs in the water samples are shown in **Fig. S2**, while the microscope images of typical NPs are shown in **Fig. S3**. Because the O-PTIR spectroscopy results corroborated the visual identification, visual sorting was used to collect at least 30 MPs and NPs from each water sample for microbiome analysis.

The MPs in each sample were quantified using chemical digestion as described previously[3]. Briefly, the particles were digested using 30% hydrogen peroxide (Sigma Aldrich, St. Louis, MO, USA) at 60°C for 24 hours and filtered through a 100-μm stainless-steel sieve. The chemical compositions of all remaining particles were analyzed using μ-Raman spectroscopy (inVia confocal Raman microscope; Renishaw, Wotton-under-Edge, UK) with a Leica 10× objective and a 785-nm excitation laser (output power: 300 mW)[4]. The Raman spectra were compared with reference spectra[4] using a non-negative least-squares algorithm to determine the polymer composition. Putative MPs with at least 70% spectral similarity to a reference were classified as that polymer type[4]. Using stereomicroscopy, the MPs were categorized into five commonly reported shapes: fragments, fibers, films, foams, and pellets[1]. The longest dimensions of the MPs were measured via stereomicroscopy, and the lengths of fibers were measured along the central axis on stereo images using ImageJ software (v.1.53t)[5]. The MPs were classified by size as 100–200, 200–300, 300–500, 500–1000, and 1000–5000 μm. The number of MPs corresponding to each polymer type, shape, and size were recorded. During MP quantification, lab blanks and recovery controls were used to ensure data reliability and monitor background contamination. Lab blanks (n = 10) consisted of 1 L of Milli-Q water, and

recovery controls included 1 L of Milli-Q water spiked with polyethylene particles: 100 particles of 50 µm (*n* = 10; Alfa Aesar, Ward Hill, MA, USA) and 50 particles of 500 µm (*n* = 10). Both underwent the same extraction, filtration, and chemical digestion as the field samples, followed by MP identification and quantification. Contamination was minimized by wearing cotton lab coats and nitrile gloves, triple-rinsing glassware with Milli-Q water, and conducting all procedures under a fume hood. No MPs (100–5,000 µm) were detected in lab blanks (**Table S2**), confirming the absence of contamination. The average recovery rates were 96.5 ± 1.1% for 50 µm particles and 96.8 ± 2.2% for 500 µm particles (**Table S2**), indicating high procedural reliability. Considering the absence of contamination and the high recovery rate, the limit of detection for MPs was estimated to be approximately 1 particle per liter to account for potential undetected losses.

Further characterization of MPs and NPs was conducted on a representative subset of visually sorted particles (MPs: *n* = 15; NPs: *n* = 15) from all sampling sites and particle types, including analysis of plastic additives, MP aging, and surface roughness. The potential presence of plastic additives on MP surfaces was assessed using O-PTIR spectroscopy with a quantum cascade laser microscope (4 cm^{-1} resolution)[6]. Specific spectral regions associated with common plastic additives such as phthalates, slip agents, hindered amine light stabilizers (HALS), and antioxidants were targeted based on previous studies[7-10]. Additive-to-reference ratios were calculated by normalizing signal intensities to invariant CH-stretch or aromatic peaks, with selected wavenumbers shown in **Fig. S6**. These intensities indicated the potential presence of the corresponding additives. O-PTIR spectroscopy was also used to assess oxidative and structural degradation of MPs due to environmental aging[11] by analyzing spectral regions associated with carbonyl formation, chain scission, and changes in crystallinity or aromatic structure, as previously identified[12-14], with corresponding wavenumbers provided in **Fig. S6**. Surface topography was examined using a KEYENCE VK-X200 3D laser microscope

(20× objective, numerical aperture 0.46, depth resolution 500 nm; KEYENCE Corporation, Itasca, IL, USA). Surface roughness was quantified using three ISO 4287 standard parameters: arithmetic mean deviation, root mean square deviation, and ten-point height.

Text S2. Metagenomic sequencing, bioinformatics processing, and diversity and indicator analyses

Genomic DNA was extracted from the identified MPs, NPs, and filters retaining RW microbes from each site using the DNeasy PowerSoil Pro Kit (Qiagen, Hilden, Germany) according to the manufacturer's instructions. Forty-five samples were processed: 15 each for the MP, NP, and RW microbiomes collected across all the study sites. Three samples containing only extraction reagents were processed in parallel as negative controls. Sequencing libraries were generated using the Rapid Plus DNA Lib Prep Kit for Illumina (ABclonal, Woburn, MA, USA) according to the manufacturer's protocol. Libraries were subjected to paired-end 150-bp sequencing on an Illumina NovaSeq 6000 platform (Novogene, Beijing, China) following the manufacturer's protocol.

Adapter removal from raw sequencing data was performed using fastp (v.0.23.1)[15] with the parameters "g -q 5 -u 50 -n 15 -l 150 --overlap_diff_limit 1 --overlap_diff_percent_limit 10." Quality filtering and trimming using KneadData (v.0.7.6) (https://huttenhower.sph.harvard.edu/kneaddata/) were then performed with the default trimmomatic options "SLIDINGWINDOW:4:20 MINLEN:50." Human sequences were then removed using KneadData, with the reference human genome hg37[16]. Species-level taxonomy was assigned to the processed reads using Kraken2 (v.2.1.2)[17] and the k2_standard_20210517_38GB database, and the relative abundance of species was quantified using Bracken (v.2.6.2)[18]. Potential contaminants were identified against the negative controls using decontam (v.1.18.0)[19] with the prevalence option under stringent mode. All eight

potential contaminant species had a low relative abundance (< 0.001%) in all field samples and were deemed insignificant for removal from the analysis. After these quality-control steps, an average of 72 ± 8.7 million high-quality paired-end reads were retained per sample. Taxonomic classification of the high-quality reads was performed using Kraken2, and read counts and relative abundances at different taxonomic ranks were calculated using Bracken.

High-quality reads from individual samples were assembled into contigs using MetaWrap (v.1.3.2)[20] with the MegaHIT module (v.1.1.3)[21] and default settings. Contigs longer than 1,000 bp were retained for functional annotation. Open reading frames (ORFs) within contigs were predicted and translated into amino acid sequences using Prodigal (v.2.6.3)[22] with the default settings. Predicted ORFs were assigned putative functions by searching against the SEED database[23] (accessed November 11, 2023), using the BLASTX function of DIAMOND (v.2.1.8)[24] with the default settings. Read counts of each ORF were calculated using the "contig" mode in CoverM (v.0.6.1, https://github.com/wwood/CoverM) with the "count" method.

Classified species and functions present in at least 20% of samples were included in composition and indicator analyses. Taxonomic and functional composition analyses were conducted using the "vegan" package (v.2.5-7) in R (v.4.1.1)[25]. For α-diversity analysis, read counts of classified species or functions with an occurrence frequency >20% in all samples were rarefied to 1×10^5 using the "rrarefy" function. Species or functional richness was calculated using the "estimateR" function, and Pielou's evenness and Shannon's diversity indexes were calculated using the "diversity" function based on the read counts of the classified species or functions. To visualize taxonomic or functional compositions in a two-dimensional ordination space, principal coordinate analysis (PCoA) was performed using the "dudi.pco" function in the R package "ade4" (v.1.7-22) based on the Bray–Curtis dissimilarity matrix of the read counts of classified species or functions. The total MP concentration and other environmental factors were incorporated into the PCoA using the "envfit" function (with 999

permutations) to determine their correlations with the taxonomic and functional compositions of the microbiomes. The relationships between pairwise taxonomic similarity (1 − Bray–Curtis dissimilarity) and pairwise similarity in the relative composition of MP types, shapes, and sizes (1 − Euclidean dissimilarity) were assessed using Mantel tests (999 permutations) via the "mantel" function in the R package "vegan." Given that the identified NPs were common natural components of rivers, their concentrations and compositions were excluded from the assessment of anthropogenic disturbances on riverine ecosystems.

Indicator analysis was used to identify taxonomic and functional indicators that could represent types of microbiomes, either alone or in combination. The strengths of associations of each species or function with specific microbiomes or groups of microbiomes were determined via correlation indices using the "multipatt" function in the R package "indicspecies" (v.1.7.14). The significance of these correlation indices was tested using 999 permutations, and p-values were adjusted using the false discovery rate (FDR) method and the "p.adjust" function in the R package "stats" (v.4.2.3). Species and functions with FDR-adjusted p-values < 0.05 were considered as representative taxonomic and functional indicators, respectively.

Text S3. Inference of life-history strategies and determination of potential ecological roles

Microbial life-history strategies were inferred from the identified functional indicators to investigate the associations of detailed life-history strategies with different types of microbiomes. The functional indicators were first grouped into ancestral classes (i.e., functional subcategories) based on hierarchical classifications from public databases (e.g., SEED, Gene Ontology, and Kyoto Encyclopedia of Genes and Genomes) or the literature. Subsequently, functional subcategories were manually classified into three main strategies—growth yield (Y), resource acquisition (A), and stress tolerance (S)—according to the trait-

based classification scheme of the Y-A-S framework as defined previously[26]. This framework reflects microbial life-history strategies in different scenarios with varying levels of resources and stresses[26,27]. Poorly classified functions were grouped under the unclassified (U) strategy. The classification results were confirmed by searching the functional indicators against a previously proposed microbial trait database[28] to classify them into Y-A-S strategies.

The potential ecological roles of MP, NP, and RW microbiomes in riverine ecosystems were represented by three important functional categories: biochemical cycling, plastic degradation, and antibiotic resistance and virulence[29-31]. To determine the metabolic capacity of the microbiomes, predicted amino acid sequences from the ORFs in assembled contigs of each sample were searched against a set of KEGG, TIGRfam, Pfam, and custom hidden Markov model profiles corresponding to key marker genes related to carbon, nitrogen, and sulfur cycling, using METABOLIC (v.4.0)[32] with the default settings. Carbohydrate-active enzymes (CAZymes) were annotated using METABOLIC based on the dbCAN2 database (dbCAN-HMMdb-V7)[33]. The presence of plastic degradation enzymes (PDEs) indicated a capacity for plastic degradation. Amino acid sequences were searched against the PlasticDB[34] and RemeDB[35] databases (both accessed May 12, 2023) using the BLASTP function of DIAMOND and the hmmscan function of HMMER (v3.1b2)[36], respectively. The results from the two databases were merged to provide comprehensive profiles of PDEs in each sample. Antibiotic resistance and virulence capacities were indicated by antibiotic resistance genes (ARGs) and virulence factors (VFs), respectively. For ARG identification, amino acid sequences were searched against the ResFams database (v.1.2)[37] using the hmmscan function of HMMER, and ARGs were classified into subtypes according to the antibiotic type to which they conferred resistance. For VF identification, amino acid sequences were searched against the full VFDB (VFDB_setB_pro) database[38] (accessed December 10, 2023) using the BLASTP function of DIAMOND; VFs were classified by pathogenesis processes as defined by the VF

database[38]. The identified putative PDE, ARG, or VF sequences were further filtered using the following cutoffs to remove low-confidence matches: E-value < 1×10^{-10}, identity > 70%, and coverage > 70% in BLASTP search; E-value < 1×10^{-10} and sequence score > 100 in hmmscan searches.

To measure the relative abundances of identified functional genes within the microbiome, gene counts were normalized to genes per million with corrections for variations in gene length and mapped reads per sample, analogous to RNA-seq transcripts per million[39]. The profiles of these important functional categories were visualized in a two-dimensional ordination space using PCoA based on the Bray–Curtis dissimilarity matrix of the relative abundances of annotated functional genes.

Text S4. Detection of potential microbial sharing and horizontal gene transfer (HGT) events

To construct metagenome-assembled genomes, contigs in each sample were clustered using MetaBAT2 (v.2.12.1)[40], Maxbin2 (v.2.2.6)[41], Concoct (v.1.0.0)[42], and SemiBin (v.1.5.1)[43]. The resulting four sets of bins were consolidated and filtered using the Bin_refinement module in MetaWrap to obtain bins with ≥ 50% completeness and ≤ 10% contamination. To obtain representative metagenome-assembled genomes (rMAGs) at the subspecies level for all samples, refined medium- or high-quality bins were dereplicated using dRep (v2.6.2)[44] with a secondary clustering threshold of 99% average nucleotide identity (ANI).

Genome-based metagenomic studies[45,46] have suggested that connected environments could harbor microbial taxa that are similar at the species level but distinct at lower taxonomic levels. Therefore, the coexistence of highly similar rMAGs at the subspecies level between MPs or NPs and RW from the same site or along the same river could be due to the colonization or spread of subspecies from connected environments, indicating potential microbial sharing

at water–particle interfaces in urban rivers. To detect the presence of rMAGs in MP, NP, and RW samples, high-quality reads from each sample were mapped to each rMAG using Bowtie2 (v.2.4.4)[47], and the resulting mappings were processed using the "inStrain profile" function in inStrain (v.1.8.0)[48] to measure genetic similarity between the rMAGs and read pairs from each sample. An rMAG was considered present in a sample if it met the following criteria: genome breadth > 0.1[49], coverage > 0.1[49], and population-level ANI > 99%[50], as determined by inStrain. Potential microbial sharing events between MPs or NPs and RW were inferred when an rMAG was shared between MP or NP and RW samples from the same site or river. In contrast, rMAGs found exclusively within a specific type of microbiome at all sites were not regarded as sharing. rMAGs that were neither shared between microbiomes at the same site or river nor specific to a particular microbiome across all sites were excluded from further analyses.

Taxonomic and functional annotations of both shared and non-shared rMAGs were performed. rMAG taxonomy was assigned using the R207 database and the "gtdbtk classify_wf" function in GTDB-Tk (v.2.0.0)[51]. A maximum likelihood phylogenetic tree of rMAGs was constructed using the "gtdbtk infer" function in GTDB-Tk and visualized using iTOL (v.6.7.3)[52]. The relative abundances of rMAGs were determined using the "genome" mode and "relative_abundance" method in CoverM. Functional and ecological role annotations were performed using the same methods applied for microbiome annotation, as indicated above. The numbers of functional genes related to ecological roles in shared and non-shared rMAGs were normalized and visualized using the "pheatmap" function in the R package "pheatmap" (v.1.0.12) with the option "scale = "column"." HGT events among shared and non-shared rMAGs were predicted using MetaCHIP (v.1.10.10)[53] with the default settings. Only HGT events among rMAGs that co-occurred within the same type of microbiome at the same site were retained.

Text S5. Data analysis and statistics

To compare environmental conditions between sampling sites, z-score-standardized measured environmental factors were subjected to principal component analysis (PCA) using the “prcomp” function in the R package “stat” (v.4.3.0). The vectors of all environmental factors were fitted onto the PCA ordination and scaled according to their PCA loadings using the “ggbiplot” function in the R package “ggbiplot” (v.0.55). The significance of the microbiome type (i.e., MP, NP, and RW) in driving variations in microbiome dissimilarity was evaluated using a permutational multivariate analysis of variance (PERMANOVA) with the “adonis” function (with 999 permutations) in the R package “vegan.” Multivariate homogeneity of group dispersions was tested via permutational analysis of multivariate dispersion (PERMDISP) using the “betadisper” and “permutest” functions (with 999 permutations) in the R package “vegan.” Pearson’s correlations of taxonomic similarity (1 − Bray–Curtis dissimilarity) with functional similarity (1 − Bray–Curtis dissimilarity) were estimated using the “cor.test” function in the R package “ggpubr” (v.0.4.0) where Pearson’s $r = 1$ indicates strict functional dependency and $r = 0$ indicates complete functional redundancy.

Differences in taxonomic and functional α-diversity, relative abundances of specific functions between any two microbiome types, as well as the extent of microbial sharing between MP and NP microbiomes, were analyzed using the two-sided Wilcoxon test with the FDR correction for multiple testing with the “wilcox_test” function in the R package “rstatix” (v.0.7.2). The linear relationships of the relative abundances of specific functions and the extent of microbial sharing with the total MP concentration were determined using the “lm” function with the FDR correction in the R package “stats”. Direct associations, controlling for other environmental factors, were further tested using partial Pearson correlation with the “pcor” function in the R package “ppcor” (v.1.1). The association between the taxonomic compositions of rMAGs and short reads, based on Bray−Curtis dissimilarity, was tested using

Procrustes analysis with the “protest” function in the R package “vegan”. Figures were created using the R package “ggplot2” (v3.3.6), except for chord diagrams, which were created using the R package “circlize” (v.0.4.15).

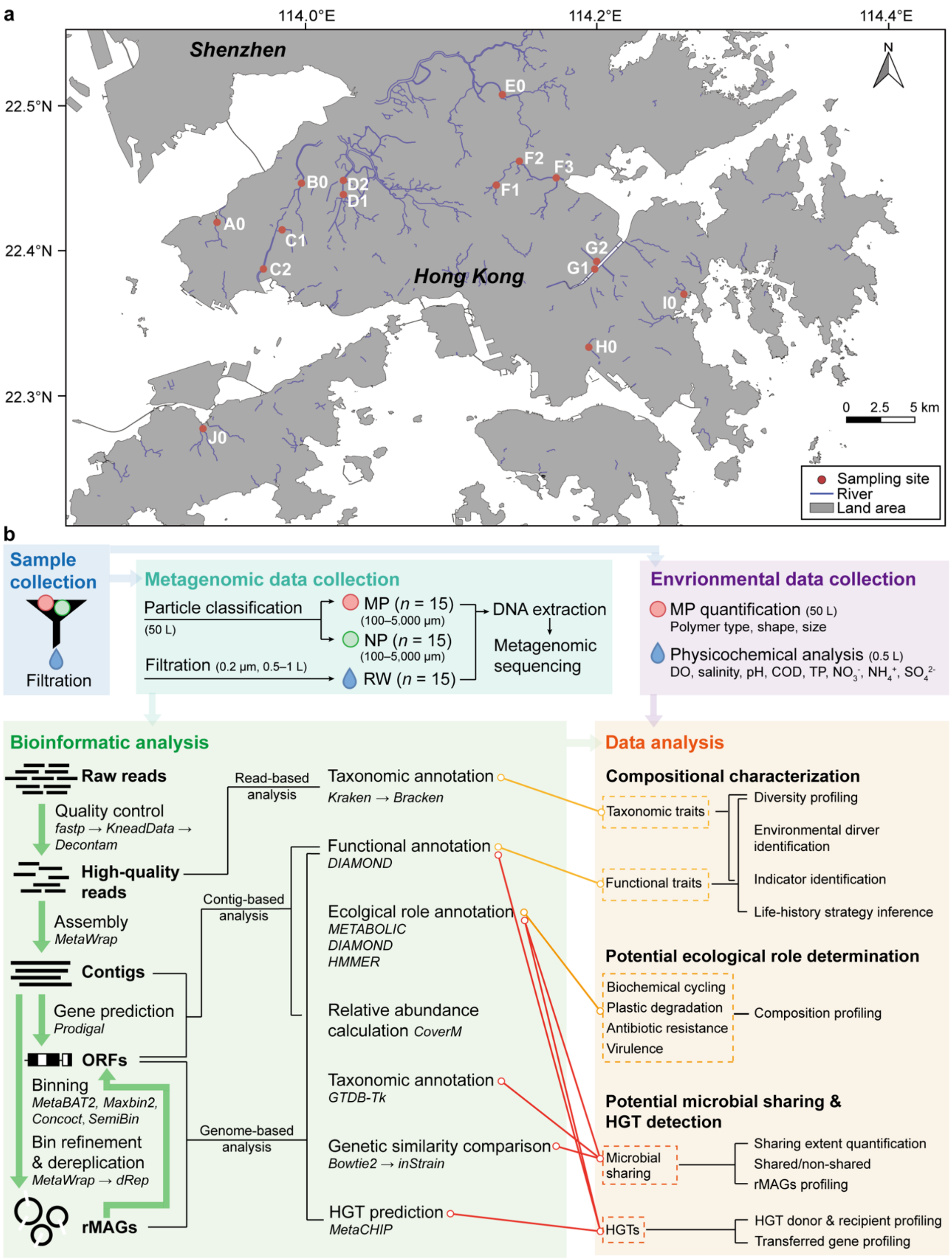

Figure S1. Overview of the sampling and analysis strategy for the study. (a) A map of the 15 sampling sites across 10 urban rivers in Hong Kong. Multiple sampling sites in the same river are labeled with numeric values increasing from upstream to downstream. **(b)** A schematic illustrating the workflow of the sampling process and key data analyses. MP: microplastics; NP: natural particles; RW: river water; DO: dissolved oxygen; COD: chemical oxygen demand; TP: total phosphorus; NO_3^-: nitrate; NH_4^+: ammonium; SO_4^{2-}: sulfate; ORFs: open reading

frames; rMAGs: representative metagenome-assembled genomes; HGTs: horizontal gene transfers.

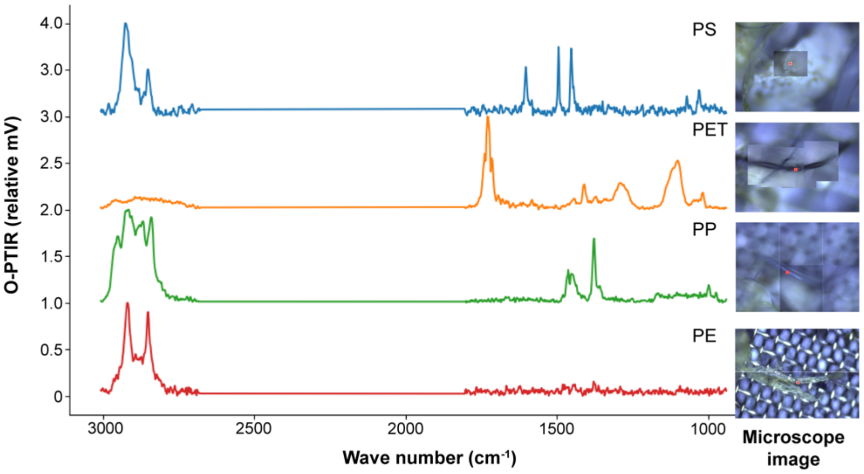

Figure S2. Representative optical photothermal infrared (O-PTIR) spectra of four polymer types of MPs in water samples. The spectra correspond to the visually identified particles shown in the microscope images on the right. PS: polystyrene; PET: polyethylene terephthalate; PP: polypropylene; PE: polyethylene.

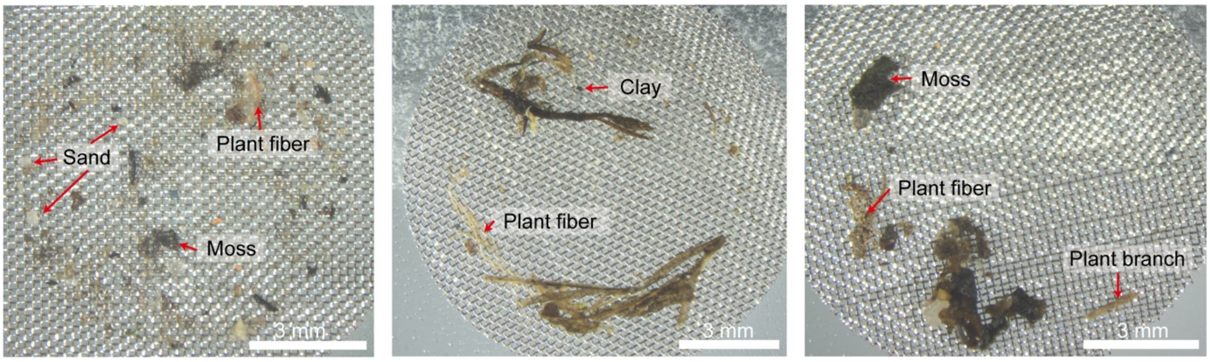

Figure S3. Representative microscopic images of different types of NPs in water samples.

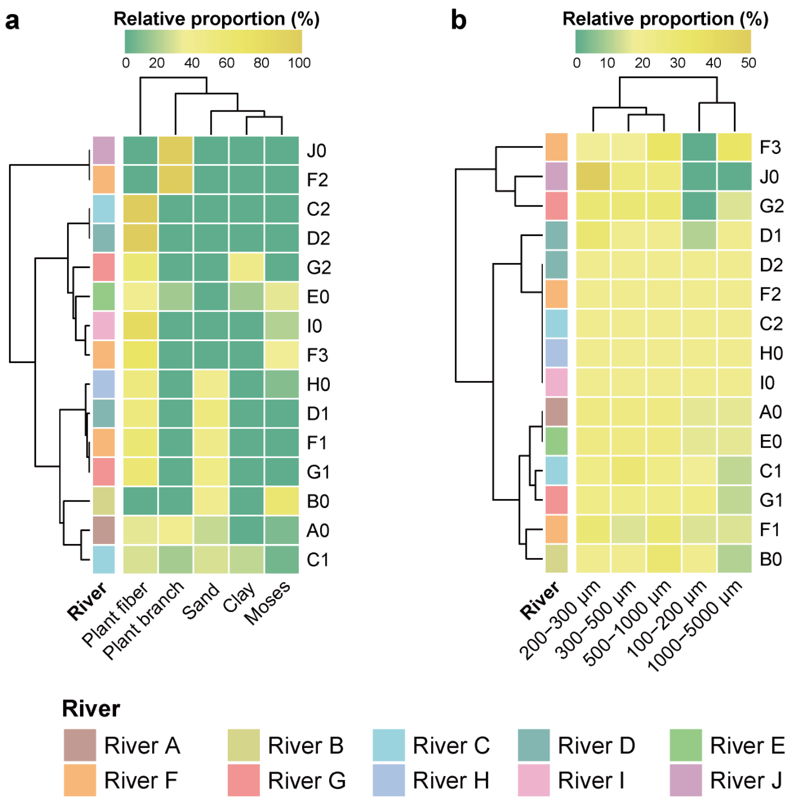

Figure S4. Hierarchical clustering heatmaps showing the relative proportions of NPs at each sampling site by (a) type and (b) size. The hierarchical tree was generated using Ward's method and Manhattan distance.

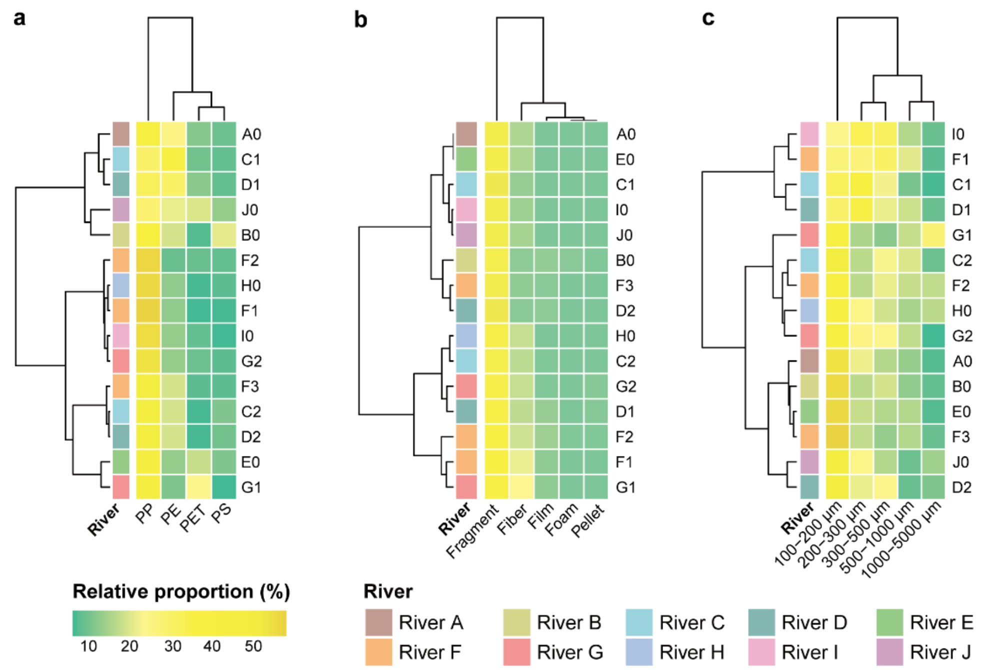

Figure S5. Hierarchical clustering heatmaps showing the relative proportions of MPs at each sampling site based on (a) polymer type, (b) shape, and (c) size. The hierarchical tree was generated using Ward's method and Manhattan distance. PS: polystyrene; PET: polyethylene terephthalate; PP: polypropylene; PE: polyethylene.

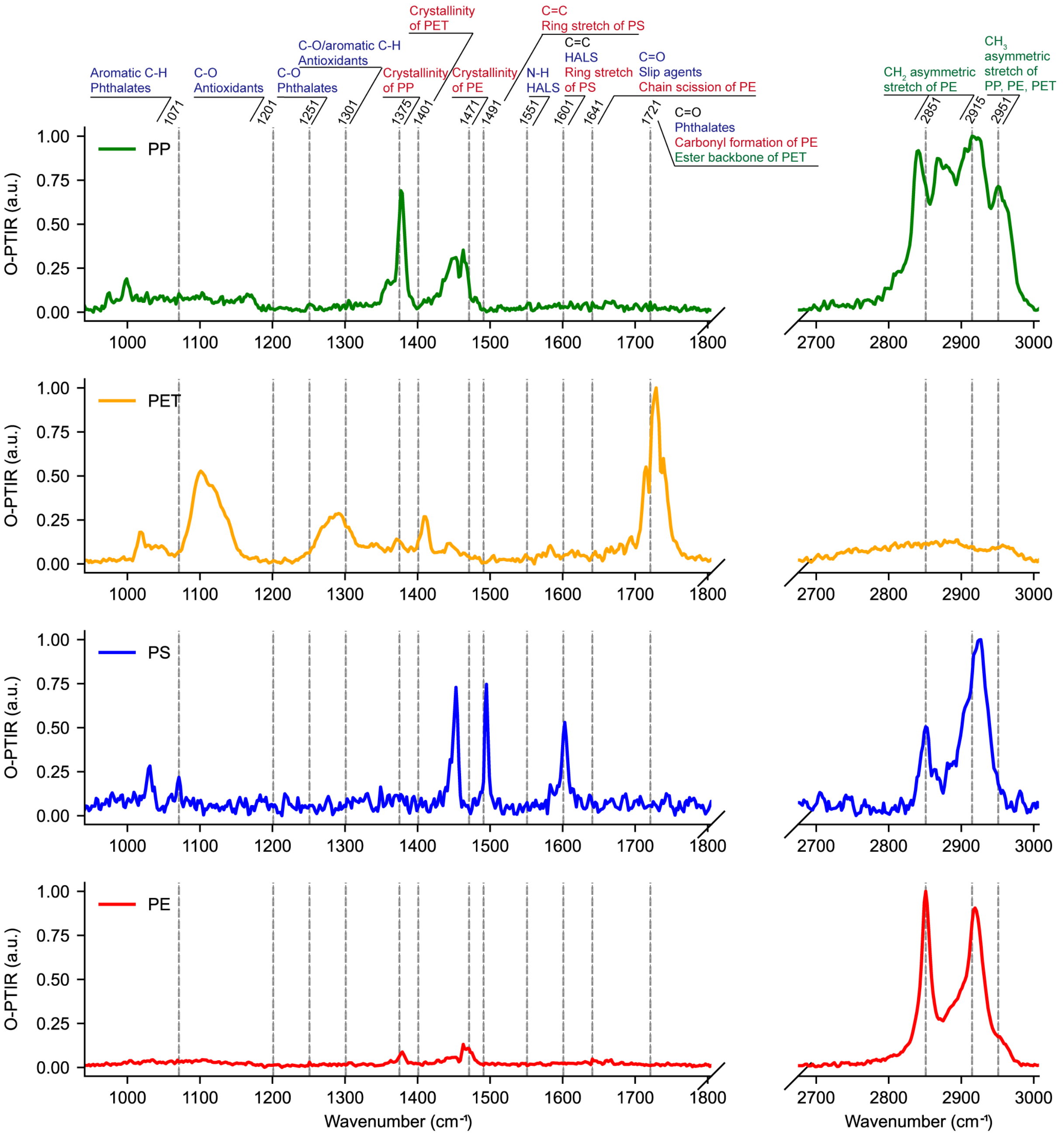

Figure S6. Representative optical photothermal infrared (O-PTIR) spectra showing potential plastic additives and signs of aging in four MP polymer types from water samples. Highlighted wavenumbers on the top of the figure indicate spectral features associated with plastic additives (blue), aging (red), and polymer-specific invariant CH-stretch or C=C ring-stretch signals (green). PS: polystyrene; PET: polyethylene terephthalate; PP: polypropylene; PE: polyethylene; HALS: hindered amine light stabilizers.

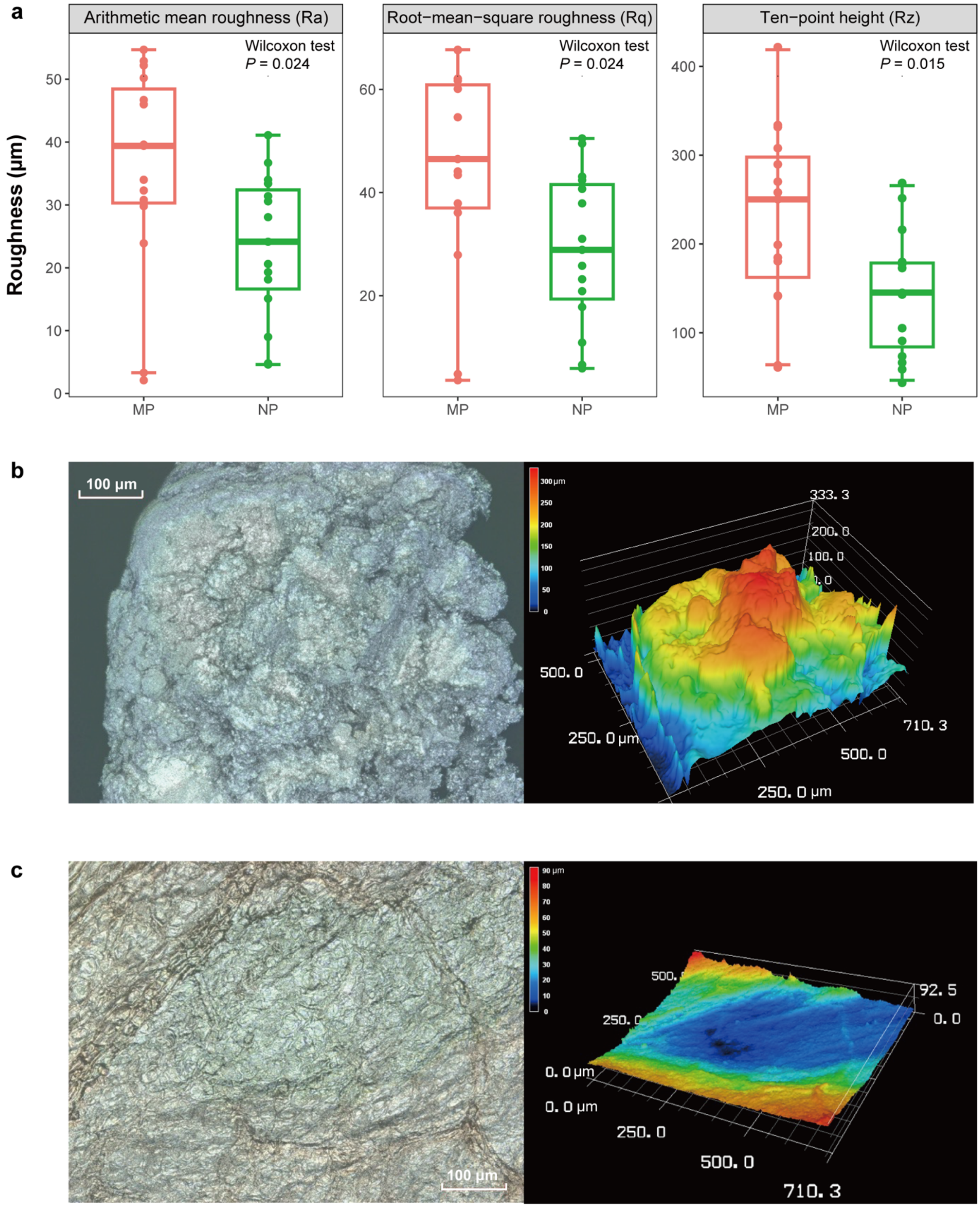

Figure S7. Surface roughness profiles of MPs and NPs. (a) Comparison of standard surface roughness parameters between MPs and NPs. (b) Representative profile of a polypropylene fragment (arithmetic mean roughness, Ra = 23.9 µm; root-mean-square roughness, Rq = 27.9 µm; and ten-point height, Rz = 143.4 µm). (c) Representative profile of a plant fiber (Ra = 15.1 µm, Rq = 17.8 µm, and Rz = 92.5 µm).

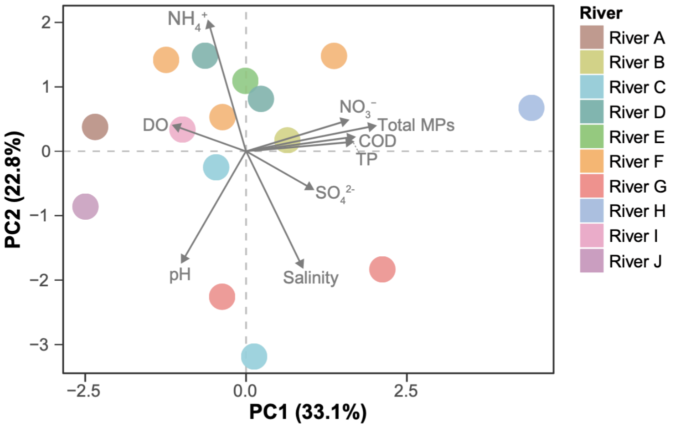

Figure S8. Principal component analysis (PCA) plot showing the nine environmental factors measured in the samples. The loadings of the environmental factors are depicted as vectors. COD: chemical oxygen demand; TP: total phosphorus; SO_4^{2-}: sulfate; DO: dissolved oxygen; NH_4^+: ammonium; NO_3^-: nitrate.

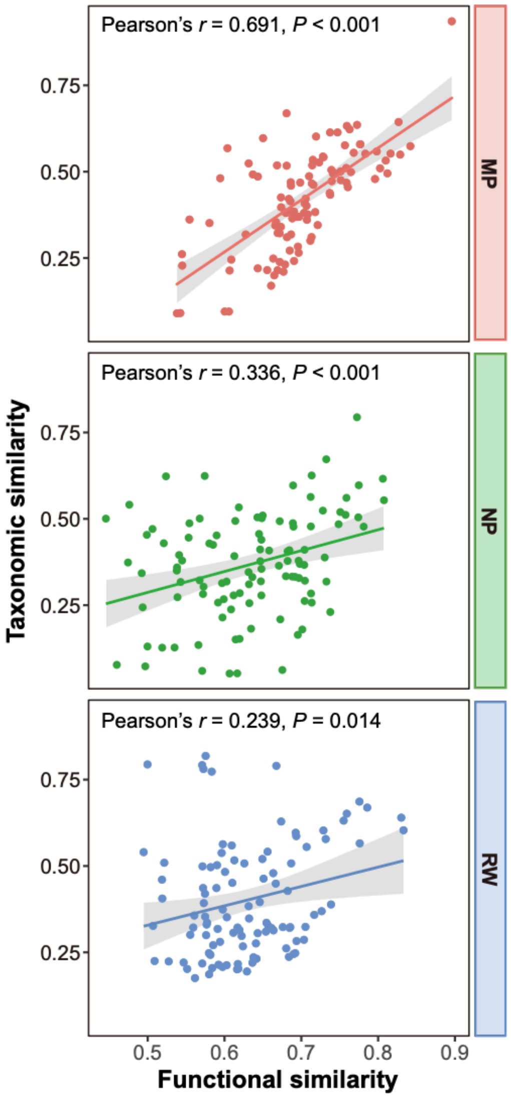

Figure S9. Relationships between functional and taxonomic similarity (measured as 1 − Bray–Curtis dissimilarity) in MP, NP, and RW microbiomes. The shaded area represents the 95% confidence interval of the linear regression.

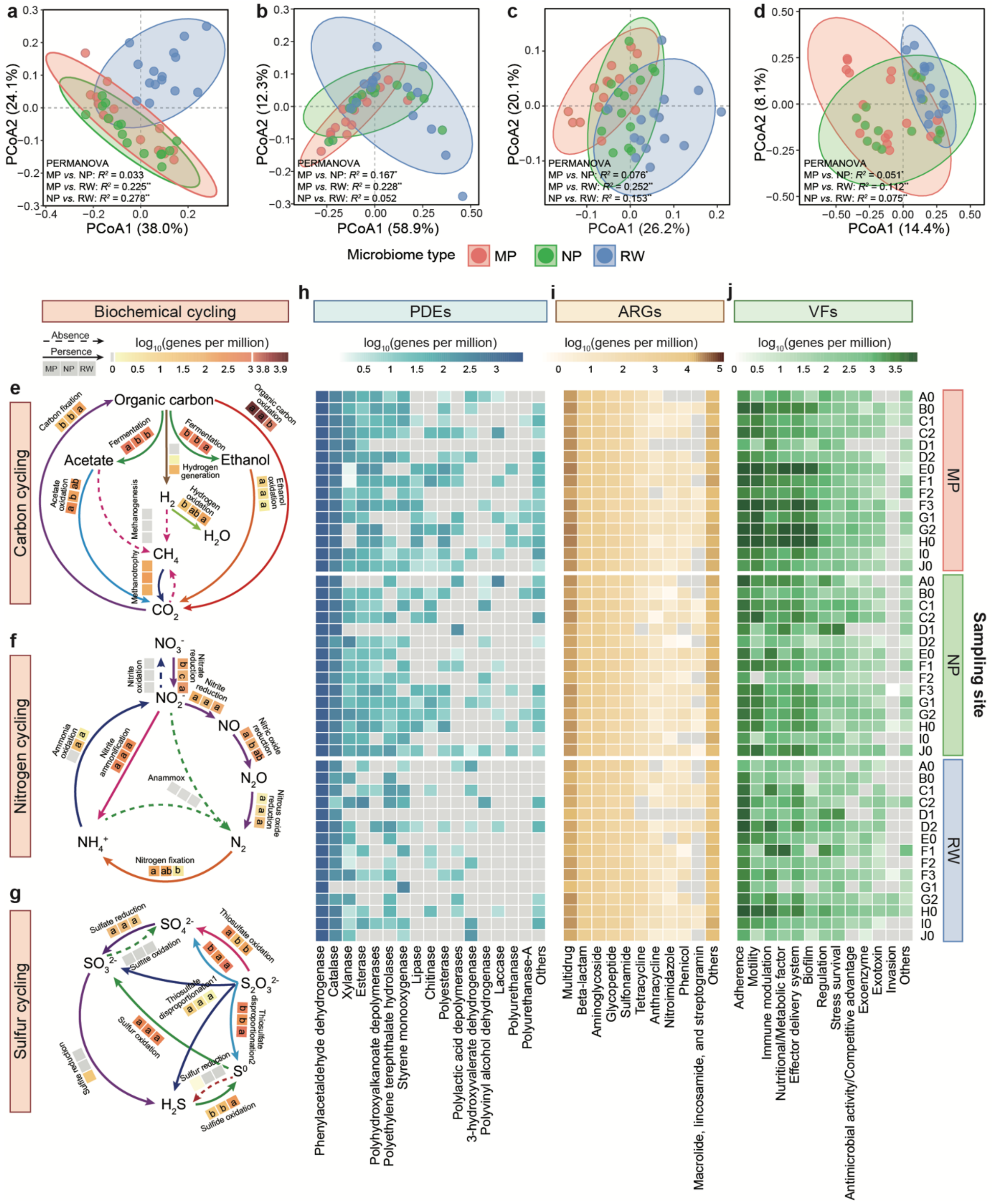

Figure S10. Ecological roles of MP, NP, and RW microbiomes. (a–d) Principal coordinate analysis (PCoA) ordination of functional compositions related to (a) biochemical cycling, (b) plastic degradation, (c) antibiotic resistance, and (d) virulence in the three types of microbiomes based on Bray–Curtis dissimilarity (** $P < 0.001$; * $P < 0.05$). Samples are colored according to the microbiome type. The ellipses represent the 90% confidence ellipse based on a multivariate t-distribution. **(e–g)** Biogeochemical cycling diagrams showing the major

metabolic processes involved in (e) carbon, (f) nitrogen, and (g) sulfur cycling. The average relative abundances of each metabolic process in MP, NP, and RW microbiomes are indicated by the direction of the arrow. The letters indicate significant differences ($P < 0.05$) between microbiomes according to the Wilcoxon test. **(h–j)** Heatmaps showing the relative abundances of the major (h) PDEs, (i) ARG subtypes, and (j) VF subcategories in the three types of microbiomes.

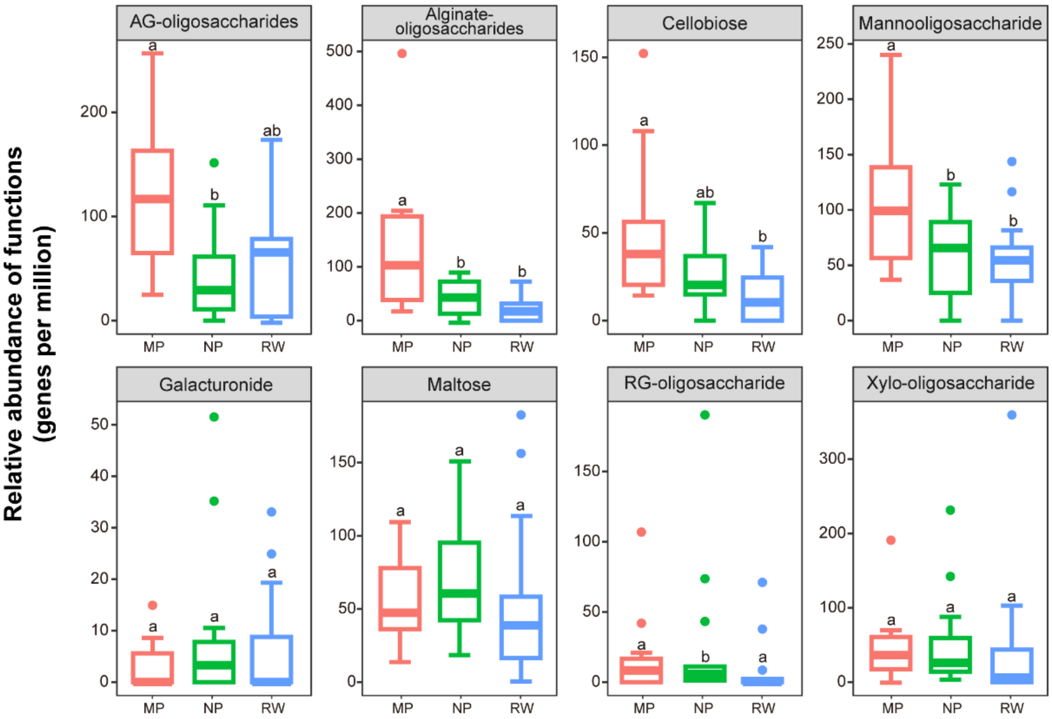

Figure S11. Relative abundances of functions related to the degradation of typical complex carbohydrates in MP, NP, and RW microbiomes. The letters indicate significant differences ($P < 0.05$) between microbiomes according to the Wilcoxon test. Each box and whiskers indicate data inside and outside the interquartile range (excluding outliers), respectively.

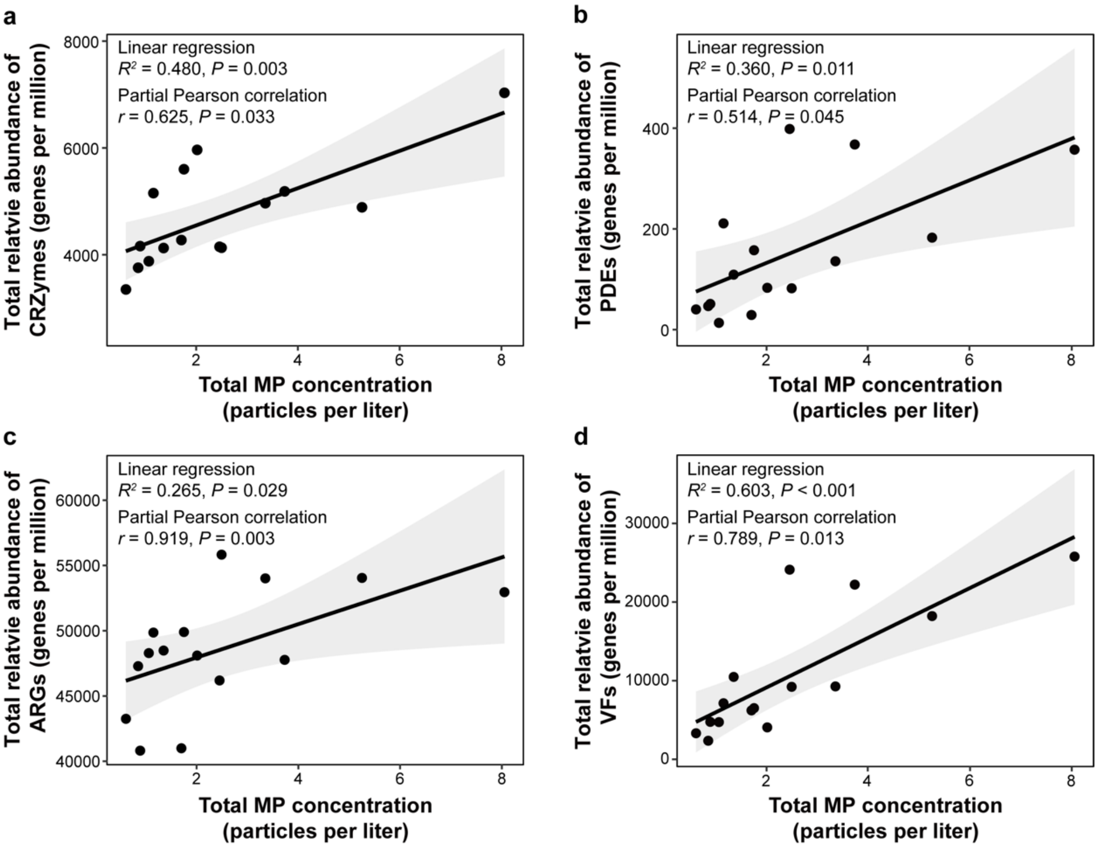

Figure S12. Relationships between the relative abundances of (a) carbohydrate-active enzymes (CAZymes), (b) plastic degradation enzymes (PDEs), (c) antibiotic resistance genes (ARGs), and (d) virulence factors (VFs) and the total MP concentration in MP microbiomes. The shaded area represents the 95% confidence interval of the linear regression.

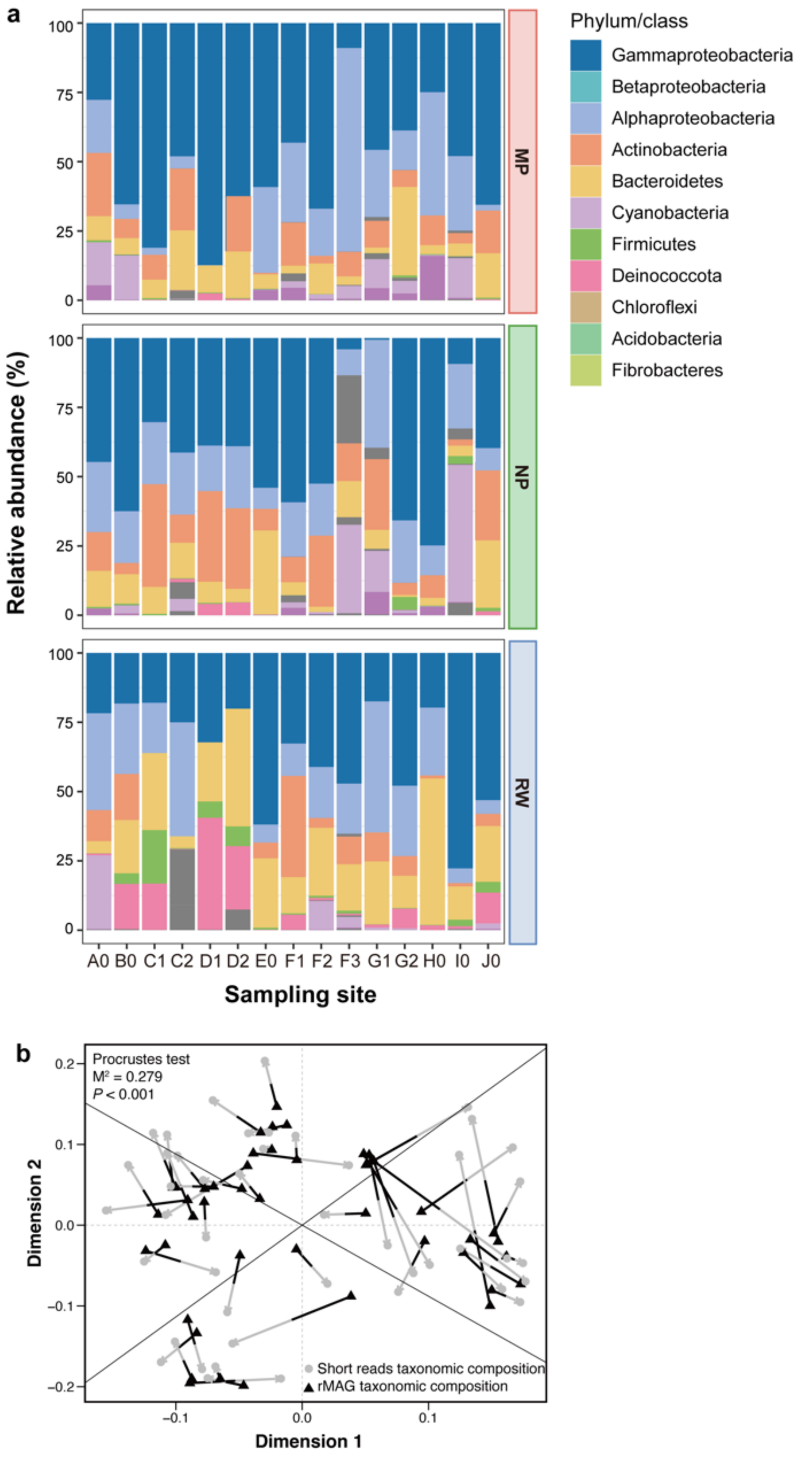

399

400 **Figure S13. (a) Relative taxonomic compositions of rMAGs at the phylum level in each**
401 **sample (class level for Proteobacteria) and (b) Procrustes analysis comparing the**
402 **microbial taxonomic compositions of rMAGs and short reads based on Bray−Curtis**
403 **dissimilarity.**

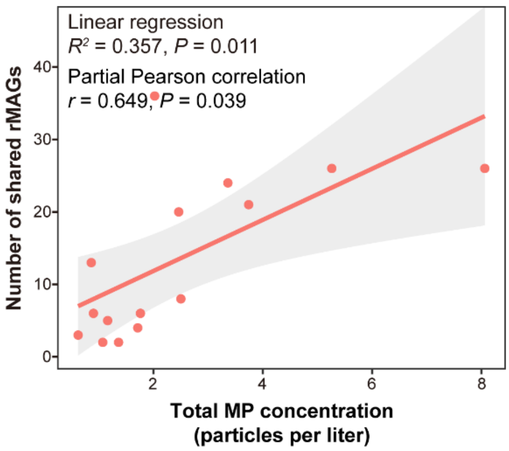

Figure S14. Relationship between the total number of shared rMAGs between MPs and the surrounding RW and the total MP concentration. The shaded area represents the 95% confidence interval of the linear regression.

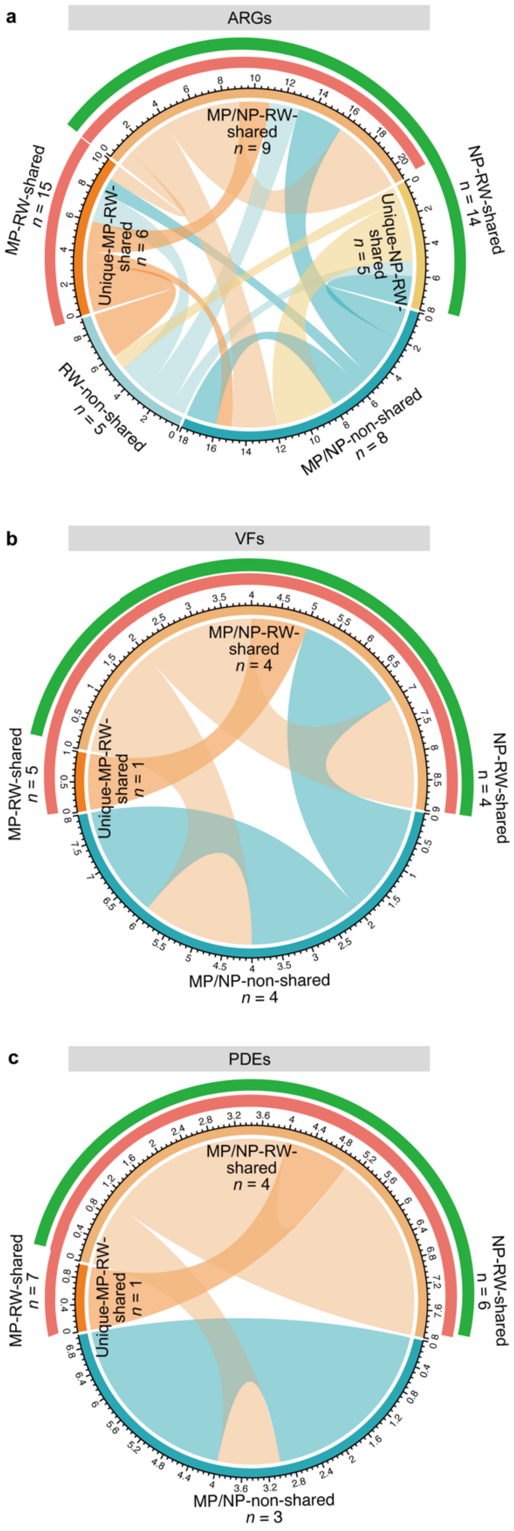

Figure S15. Predicted HGTs of (a) antibiotic resistance genes (ARGs), (b) virulence factors (VFs), and (c) putative plastic degradation enzymes (PDEs) among shared and non-shared rMAGs. Each band connects the HGT donor with its recipient; the width of the band represents the number of HGT events, and the color represents the HGT donor.

References

(1) Hidalgo-Ruz, V.; Gutow, L.; Thompson, R. C.; Thiel, M., Microplastics in the marine environment: a review of the methods used for identification and quantification. *Environ. Sci. Technol.* **2012,** *46*, (6), 3060-3075.
(2) Kansiz, M.; Prater, C.; Dillon, E.; Lo, M.; Anderson, J.; Marcott, C.; Demissie, A.; Chen, Y.; Kunkel, G., Optical photothermal infrared microspectroscopy with simultaneous Raman–a new non-contact failure analysis technique for identification of< 10 μm organic contamination in the hard drive and other electronics industries. *Microscopy today* **2020,** *28*, (3), 26-36.
(3) Li, J.; Qu, X.; Su, L.; Zhang, W.; Yang, D.; Kolandhasamy, P.; Li, D.; Shi, H., Microplastics in mussels along the coastal waters of China. *Environ. Pollut.* **2016,** *214*, 177-184.
(4) Leung, M. M.-L.; Ho, Y.-W.; Maboloc, E. A.; Lee, C.-H.; Wang, Y.; Hu, M.; Cheung, S.-G.; Fang, J. K.-H., Determination of microplastics in the edible green-lipped mussel Perna viridis using an automated mapping technique of Raman microspectroscopy. *J. Hazard. Mater.* **2021,** *420*, 126541.
(5) Schneider, C. A.; Rasband, W. S.; Eliceiri, K. W., NIH Image to ImageJ: 25 years of image analysis. *Nat. Methods* **2012,** *9*, (7), 671-675.
(6) Su, Y.; Hu, X.; Tang, H.; Lu, K.; Li, H.; Liu, S.; Xing, B.; Ji, R., Steam disinfection releases micro (nano) plastics from silicone-rubber baby teats as examined by optical photothermal infrared microspectroscopy. *Nat. Nanotechnol.* **2022,** *17*, (1), 76-85.
(7) Verleye, G. A.; Roeges, N. P.; De Moor, M. O., *Easy identification of plastics and rubbers*. iSmithers Rapra Publishing: 2001.
(8) Socrates, G., *Infrared and Raman characteristic group frequencies: tables and charts*. John Wiley & Sons: 2004.
(9) Mecozzi, M.; Pietroletti, M.; Monakhova, Y. B., FTIR spectroscopy supported by statistical techniques for the structural characterization of plastic debris in the marine environment: application to monitoring studies. *Mar. Pollut. Bull.* **2016,** *106*, (1-2), 155-161.
(10) Hummel, D. O., *Atlas of plastics additives: analysis by spectrometric methods*. Springer Science & Business Media: 2002.
(11) Zhou, T.; Wu, J.; Liu, Y.; Xu, A., Seawater accelerated the aging of polystyrene and enhanced its toxic effects on Caenorhabditis elegans. *Int. J. Mol. Sci.* **2023,** *24*, (24), 17219.
(12) Chen, X.; Xu, M.; Yuan, L.-m.; Huang, G.; Chen, X.; Shi, W., Degradation degree analysis of environmental microplastics by micro FT-IR imaging technology. *Chemosphere* **2021,** *274*, 129779.
(13) Bhat, M.; Gedik, K.; Gaga, E., A preliminary study on the natural aging behavior of microplastics in indoor and outdoor environments. *Int. J. Environ. Sci. Technol.* **2024,** *21*, (2), 1923-1936.
(14) Veerasingam, S.; Ranjani, M.; Venkatachalapathy, R.; Bagaev, A.; Mukhanov, V.; Litvinyuk, D.; Mugilarasan, M.; Gurumoorthi, K.; Guganathan, L.; Aboobacker, V., Contributions of Fourier transform infrared spectroscopy in microplastic pollution research: A review. *Crit. Rev. Environ. Sci. Technol.* **2021,** *51*, (22), 2681-2743.
(15) Chen, S., Ultrafast one-pass FASTQ data preprocessing, quality control, and deduplication using fastp. *Imeta* **2023,** *2*, (2), e107.
(16) McIver, L. J.; Abu-Ali, G.; Franzosa, E. A.; Schwager, R.; Morgan, X. C.; Waldron, L.; Segata, N.; Huttenhower, C., bioBakery: a meta'omic analysis environment. *Bioinformatics*

2018, *34*, (7), 1235–1237.
(17) Wood, D. E.; Lu, J.; Langmead, B., Improved metagenomic analysis with Kraken 2. *Genome Biol.* **2019,** *20*, (1), 257.
(18) Lu, J.; Rincon, N.; Wood, D. E.; Breitwieser, F. P.; Pockrandt, C.; Langmead, B.; Salzberg, S. L.; Steinegger, M., Metagenome analysis using the Kraken software suite. *Nat. Protoc.* **2022,** *17*, (12), 2815-2839.
(19) Davis, N. M.; Proctor, D. M.; Holmes, S. P.; Relman, D. A.; Callahan, B. J., Simple statistical identification and removal of contaminant sequences in marker-gene and metagenomics data. *Microbiome* **2018,** *6*, (1), 226.
(20) Uritskiy, G. V.; DiRuggiero, J.; Taylor, J., MetaWRAP—a flexible pipeline for genome-resolved metagenomic data analysis. *Microbiome* **2018,** *6*, (1), 1-13.
(21) Li, D.; Liu, C. M.; Luo, R.; Sadakane, K.; Lam, T. W., MEGAHIT: an ultra-fast single-node solution for large and complex metagenomics assembly via succinct de Bruijn graph. *Bioinformatics* **2015,** *31*, (10), 1674-6.
(22) Hyatt, D.; Chen, G.-L.; LoCascio, P. F.; Land, M. L.; Larimer, F. W.; Hauser, L. J., Prodigal: prokaryotic gene recognition and translation initiation site identification. *BMC Bioinformatics* **2010,** *11*, 1-11.
(23) Overbeek, R.; Olson, R.; Pusch, G. D.; Olsen, G. J.; Davis, J. J.; Disz, T.; Edwards, R. A.; Gerdes, S.; Parrello, B.; Shukla, M.; Vonstein, V.; Wattam, A. R.; Xia, F.; Stevens, R., The SEED and the Rapid Annotation of microbial genomes using Subsystems Technology (RAST). *Nucleic Acids Res.* **2014,** *42*, (Database issue), D206-14.
(24) Buchfink, B.; Reuter, K.; Drost, H.-G., Sensitive protein alignments at tree-of-life scale using DIAMOND. *Nat. Methods* **2021,** *18*, (4), 366-368.
(25) Team, R. C., R: A language and environment for statistical computing. *R Foundation for Statistical Computing, Vienna.* **2022**.
(26) Malik, A. A.; Martiny, J. B. H.; Brodie, E. L.; Martiny, A. C.; Treseder, K. K.; Allison, S. D., Defining trait-based microbial strategies with consequences for soil carbon cycling under climate change. *ISME J.* **2020,** *14*, (1), 1-9.
(27) Li, C.; Liao, H.; Xu, L.; Wang, C.; He, N.; Wang, J.; Li, X., The adjustment of life history strategies drives the ecological adaptations of soil microbiota to aridity. *Mol. Ecol.* **2022,** *31*, (10), 2920-2934.
(28) Karaoz, U.; Brodie, E. L., microTrait: A Toolset for a Trait-Based Representation of Microbial Genomes. *Front. Bioinform.* **2022,** *2*, 918853.
(29) Battin, T. J.; Besemer, K.; Bengtsson, M. M.; Romani, A. M.; Packmann, A. I., The ecology and biogeochemistry of stream biofilms. *Nat. Rev. Microbiol.* **2016,** *14*, (4), 251-263.
(30) Li, R.; Zhu, L.; Wang, Y.; Zhu, Y.-G., Metagenomic insights into environmental risk of field microplastics in an urban river. *Water Res.* **2022,** *223*, 119018.
(31) Wright, R. J.; Bosch, R.; Langille, M. G. I.; Gibson, M. I.; Christie-Oleza, J. A., A multi-OMIC characterisation of biodegradation and microbial community succession within the PET plastisphere. *Microbiome* **2021,** *9*, (1), 141.
(32) Zhou, Z.; Tran, P. Q.; Breister, A. M.; Liu, Y.; Kieft, K.; Cowley, E. S.; Karaoz, U.; Anantharaman, K., METABOLIC: high-throughput profiling of microbial genomes for functional traits, metabolism, biogeochemistry, and community-scale functional networks. *Microbiome* **2022,** *10*, (1), 33.

(33) Yin, Y.; Mao, X.; Yang, J.; Chen, X.; Mao, F.; Xu, Y., dbCAN: a web resource for automated carbohydrate-active enzyme annotation. *Nucleic Acids Res.* **2012,** *40*, (W1), W445-W451.

(34) Gambarini, V.; Pantos, O.; Kingsbury, J. M.; Weaver, L.; Handley, K. M.; Lear, G., PlasticDB: a database of microorganisms and proteins linked to plastic biodegradation. *Database* **2022,** *2022*, baac008.

(35) Sankara Subramanian, S. H.; Balachandran, K. R. S.; Rangamaran, V. R.; Gopal, D., RemeDB: Tool for Rapid Prediction of Enzymes Involved in Bioremediation from High-Throughput Metagenome Data Sets. *J. Comput. Biol.* **2020,** *27*, (7), 1020-1029.

(36) Finn, R. D.; Clements, J.; Eddy, S. R., HMMER web server: interactive sequence similarity searching. *Nucleic Acids Res.* **2011,** *39*, (Web Server issue), W29-37.

(37) Gibson, M. K.; Forsberg, K. J.; Dantas, G., Improved annotation of antibiotic resistance determinants reveals microbial resistomes cluster by ecology. *ISME J.* **2015,** *9*, (1), 207-16.

(38) Liu, B.; Zheng, D.; Zhou, S.; Chen, L.; Yang, J., VFDB 2022: a general classification scheme for bacterial virulence factors. *Nucleic Acids Res.* **2021,** *50*, (D1), D912-D917.

(39) Wagner, G. P.; Kin, K.; Lynch, V. J., Measurement of mRNA abundance using RNA-seq data: RPKM measure is inconsistent among samples. *Theory Biosci.* **2012,** *131*, (4), 281-5.

(40) Kang, D. D.; Li, F.; Kirton, E.; Thomas, A.; Egan, R.; An, H.; Wang, Z., MetaBAT 2: an adaptive binning algorithm for robust and efficient genome reconstruction from metagenome assemblies. *PeerJ* **2019,** *7*, e7359.

(41) Wu, Y.-W.; Simmons, B. A.; Singer, S. W., MaxBin 2.0: an automated binning algorithm to recover genomes from multiple metagenomic datasets. *Bioinformatics* **2015,** *32*, (4), 605-607.

(42) Alneberg, J.; Bjarnason, B. S.; de Bruijn, I.; Schirmer, M.; Quick, J.; Ijaz, U. Z.; Lahti, L.; Loman, N. J.; Andersson, A. F.; Quince, C., Binning metagenomic contigs by coverage and composition. *Nat. Methods* **2014,** *11*, (11), 1144-1146.

(43) Pan, S.; Zhu, C.; Zhao, X. M.; Coelho, L. P., A deep siamese neural network improves metagenome-assembled genomes in microbiome datasets across different environments. *Nat. Commun.* **2022,** *13*, (1), 2326.

(44) Olm, M. R.; Brown, C. T.; Brooks, B.; Banfield, J. F., dRep: a tool for fast and accurate genomic comparisons that enables improved genome recovery from metagenomes through de-replication. *ISME J.* **2017,** *11*, (12), 2864-2868.

(45) Raveh-Sadka, T.; Firek, B.; Sharon, I.; Baker, R.; Brown, C. T.; Thomas, B. C.; Morowitz, M. J.; Banfield, J. F., Evidence for persistent and shared bacterial strains against a background of largely unique gut colonization in hospitalized premature infants. *ISME J.* **2016,** *10*, (12), 2817-2830.

(46) Raveh-Sadka, T.; Thomas, B. C.; Singh, A.; Firek, B.; Brooks, B.; Castelle, C. J.; Sharon, I.; Baker, R.; Good, M.; Morowitz, M. J.; Banfield, J. F., Gut bacteria are rarely shared by co-hospitalized premature infants, regardless of necrotizing enterocolitis development. *Elife* **2015,** *4*, e05477.

(47) Langmead, B.; Salzberg, S. L., Fast gapped-read alignment with Bowtie 2. *Nat. Methods* **2012,** *9*, (4), 357-9.

(48) Olm, M. R.; Crits-Christoph, A.; Bouma-Gregson, K.; Firek, B. A.; Morowitz, M. J.; Banfield, J. F., inStrain profiles population microdiversity from metagenomic data and

sensitively detects shared microbial strains. *Nat. Biotechnol.* **2021,** *39*, (6), 727-736.
(49) Carter, M. M.; Olm, M. R.; Merrill, B. D.; Dahan, D.; Tripathi, S.; Spencer, S. P.; Yu, F. B.; Jain, S.; Neff, N.; Jha, A. R.; Sonnenburg, E. D.; Sonnenburg, J. L., Ultra-deep sequencing of Hadza hunter-gatherers recovers vanishing gut microbes. *Cell* **2023,** *186*, (14), 3111-3124.e13.
(50) Brooks, B.; Olm, M. R.; Firek, B. A.; Baker, R.; Thomas, B. C.; Morowitz, M. J.; Banfield, J. F., Strain-resolved analysis of hospital rooms and infants reveals overlap between the human and room microbiome. *Nat. Commun.* **2017,** *8*, (1), 1814.
(51) Chaumeil, P.-A.; Mussig, A. J.; Hugenholtz, P.; Parks, D. H., GTDB-Tk v2: memory friendly classification with the genome taxonomy database. *Bioinformatics* **2022,** *38*, (23), 5315-5316.
(52) Pedregosa, F.; Varoquaux, G.; Gramfort, A.; Michel, V.; Thirion, B.; Grisel, O.; Blondel, M.; Prettenhofer, P.; Weiss, R.; Dubourg, V., Scikit-learn: Machine learning in Python. *J. Mach. Learn. Res.* **2011,** *12*, 2825-2830.
(53) Song, W.; Wemheuer, B.; Zhang, S.; Steensen, K.; Thomas, T., MetaCHIP: community-level horizontal gene transfer identification through the combination of best-match and phylogenetic approaches. *Microbiome* **2019,** *7*, (1), 36.
